# Deficiency of the mitochondrial transporter SLC25A47 minimally impacts hepatic lipid metabolism in fasted and diet-induced obese mice

**DOI:** 10.1016/j.molmet.2024.102092

**Published:** 2024-12-31

**Authors:** Brecht Attema, Montserrat A. de la Rosa Rodriguez, Evert M. van Schothorst, Sander Grefte, Guido JEJ. Hooiveld, Sander Kersten

**Affiliations:** 1 Nutrition, Metabolism and Genomics Group, Division of Human Nutrition and Health, Wageningen University, Stippeneng 4, 6708 WE Wageningen, the Netherlands; 2 Human and Animal Physiology, Wageningen University, Wageningen, the Netherlands; 3 Division of Nutritional Sciences, Cornell University, Ithaca, NY 14853, USA

**Keywords:** Liver, Fasting, PPARα, SLC25A47, Mitochondria, Fatty acids, Triglycerides

## Abstract

**Objective:**

The peroxisome proliferator-activated receptor-alpha (PPARα) plays a central role in lipid metabolism in the liver by stimulating the expression of hundreds of genes. Accordingly, regulation by PPARα could be a screening tool to identify novel genes involved in hepatic lipid metabolism. Previously, the mitochondrial transporter SLC25A47 was suggested to play a role in energy metabolism and liver-specific uncoupling, but further research is lacking.

**Methods:**

We explored the potential role of SLC25A47 through in vitro studies and using mice overexpressing and lacking SLC25A47.

**Results:**

*SLC25A47* was identified as a PPARα-regulated and fasting-induced gene in human and mouse hepatocytes. Adenoviral-mediated overexpression of SLC25A47 minimally impacted metabolic parameters during fasting and high-fat feeding. During high-fat feeding, SLC25A47 ablation also did not influence any metabolic parameters, apart from a minor improvement in glucose tolerance. In fasted mice, SLC25A47 ablation was associated with modest, reproducible, and likely indirect reductions in plasma triglycerides and glycerol. SLC25A47 ablation did not influence energy expenditure. Depending on the nutritional status, metabolomic analysis showed modest alterations in plasma, liver, and hepatic mitochondrial levels of various metabolites related to amino acid metabolism, TCA cycle, and fatty acid metabolism. No major and consistent alterations in levels of specific metabolites were found that establish the substrate for and function of SLC25A47.

**Conclusion:**

Collectively, our results hint at a role of SLC25A47 in amino acid and fatty acid metabolism, yet suggest that SLC25A47 is dispensable for hepatic lipid homeostasis during fasting and high-fat feeding.

## Highlights

• Expression of SLC25A47 is regulated by PPARα in mouse and human hepatocytes.
• *Slc25a47*^*−/−*^ mice display modest reductions in plasma triglycerides during fasting.
• Body weight, energy expenditure and mito function are not affected in *Slc25a47*^*−/−*^ mice during fasting or high-fat feeding.
• Studies in mice suggest a redundant role of SLC25A47 in hepatic lipid and amino acid metabolism.

## 1 Introduction

The liver plays a central role in governing lipid metabolism in various nutritional conditions. In the post-prandial state, the liver actively synthesizes cholesterol, bile acids, and fatty acids. In the fasted state, the liver takes up large quantities of adipose tissue-derived fatty acids, which are either oxidized to CO_2_, converted into ketone bodies, or esterified into triglycerides [1]. The latter are stored in lipid droplets or secreted as a component of very low-density lipoproteins [2].

The changes in lipid metabolism during feeding and fasting are partly driven by changes in circulating levels of metabolic hormones and are largely effectuated through enzyme regulation at the transcriptional, translational, and post-translational levels. A group of ligand-activated transcription factors that play a central role in the regulation of lipid metabolism are the peroxisome proliferator-activated receptors (PPARs) [3]. PPARs are activated by a variety of fatty acids and fatty acid-derived compounds, as well as by certain drugs and other synthetic compounds. Three different PPARs can be distinguished: PPARα (Nr1c1), PPARβ/δ (Nr1c2), and PPARγ (Nr1c3) [3]. Although all PPARs are expressed to some extent in the liver, PPARα is considered the dominant isotype in the liver. Transcriptome analyses of the liver of whole-body and liver-specific PPARα^−/−^ mice have demonstrated that PPARα induces the expression of hundreds of genes involved in the hepatic processing of lipids, including fatty acid uptake, transport, activation, elongation, esterification, storage, and oxidation [[4], [5], [6]]. The role of PPARα as a transcriptional regulator of hepatic lipid metabolism is most prominent in the fasted state [[4], [5], [6], [7], [8]], indicating a primary role for PPARα in the adaptive response to fasting.

Studies in human hepatocytes, human liver slices, humanized PPARα mice, and hepatocyte humanized mice support the major role of PPARα in regulating hepatic lipid metabolism in human hepatocytes but also underscore the more modest effect of PPARα activation on gene regulation in the human liver compared to mouse liver [[9], [10], [11], [12], [13]]. In agreement with this notion, it was observed in hepatocyte humanized mice that the upregulation of genes involved in various PPARα-dependent pathways of fatty acid catabolism and storage by fasting was generally more modest in human liver cells than in mouse liver cells [1]. Nevertheless, many genes are consistently induced by fasting and PPARα in mouse and human hepatocytes, including *ACOX1*, *HMGCS2*, *CPT1A*, *APOA5*, and *ANGPTL4*. Considering that most of the genes induced by PPARα are involved in lipid homeostasis, regulation by PPARα could be used as a screening tool to identify novel genes involved in hepatic lipid metabolism.

In this paper, we describe the identification of the mitochondrial transporter SLC25A47 as a PPARα-regulated gene in human and mouse hepatocytes. SLC25A47 is a member of the family of six-transmembrane-helix mitochondrial SLC25 (solute carrier family 25) transporters [[14], [15], [16]]. These carriers facilitate the transport across the inner mitochondrial membrane of a chemically diverse set of solutes, including amino acids, acyl-carnitine, nucleotides, and protons. Based on a similar sequence and structural properties, 53 members of the SLC25 family have been identified in the human genome, including several orphan transporters [[14], [15], [16]]. SLC25A47 was previously proposed as a liver-specific mitochondrial uncoupling protein [17,18]. As our studies were underway, several papers were published proposing a role for SLC25A47 in fatty acid oxidation, gluconeogenesis, and nicotinamide adenine dinucleotide (NAD^+^) transport [[19], [20], [21]]. Here, we present the collective data from our in vivo and in vitro studies aimed at exploring the potential function of SLC25A47 in hepatic metabolism. Overall, we do not confirm the uncoupling properties of SLC25A47. Overexpression of SLC25A47 did not have noticeable effects on hepatic and whole-body metabolism, either during fasting or in diet-induced obese mice. Similarly, the metabolic effects of SLC25A47 deficiency during fasting or after feeding different types of high-fat diets were minor. However, we did find modest, reproducible, and likely indirect reductions in fasting plasma triglycerides and glycerol in SLC25A47-deficient mice. In addition, SLC25A47 deficiency was associated with improved glucose tolerance in diet-induced obese mice. We did not find any major and consistent alterations in levels of specific metabolites that might point at the substrate for SLC25A47. Even though our studies were not specifically set up to reproduce the results and validate the conclusions reached in the other publications [[19], [20], [21]], we do not confirm an effect of SLC25A47 ablation on body weight, mitochondrial stress, fibrosis, gluconeogenesis, uncoupling, or energy expenditure. Collectively, our results suggest that SLC25A47 is dispensable for hepatic lipid homeostasis during fasting and high-fat feeding.

## 2 Methods

### 2.1 Animals

During the various interventions, the mice were housed at 21–22 °C under specific pathogen–free conditions and followed a 6:00–18:00 day–night cycle. Mice were fed a standard chow diet after weaning. Unless otherwise indicated, mice had ad libitum access to food and drinking water during the study. During fasting experiments, mice had ad libitum access to drinking water. At the end of the studies, mice were euthanized between 8.30 and 10.00 in the morning. The mice were first anesthetized with a mixture of isoflurane (1.5%), nitrous oxide (70%), and oxygen (30%), followed by the collection of blood into EDTA tubes via orbital puncture. Mice were euthanized by cervical dislocation, after which tissues were excised and snap-frozen in liquid nitrogen.

SLC25A47-mutant mice (Slc25a47tm1a(EUCOMM)Hmgu, on C57BL/6N background) were acquired from the EUCOMM/KOMP repository. Mice heterozygous for the initial allele (Tm1a) were cross-bred with transgenic mice in which Cre recombinase is expressed in hepatocytes under the control of the Albumin gene promoter (Albumin-Cre, B6.Cg-Speer6-ps1^Tg(Alb-cre)21Mgn^/J, #003574, on C57BL/6J background; Jackson Laboratories, Bar Harbor, ME), leading over several generations to the generation of homozygous Tm1a and Tm1b mice. The first large study with these mice (indirect calorimetry followed by low- or high-fat feeding) was conducted comparing wild-type and Tm1b littermates obtained through heterozygous breedings. However, we later found that Tm1a mice do not produce any *Slc25a4*7 mRNA (Sup Figure 1). Therefore, in all subsequent studies, the Tm1a and Tm1b mice were grouped and referred to as *Slc25a47*^*−/−*^ mice.

The animal studies were all carried out at the Centre for Small Animals, which is part of the Centralized Facilities for Animal Research at Wageningen University and Research (CARUS), and were approved by the Central Committee for Animal Experimentation (CCD) (AVD104002015236, AVD10400202115283) and the Local Animal Ethics Committee of Wageningen University (2014091.b, 2016015.e, 2016.W-0093.012, 2016.W-0093.016, 2016.W-0093.022, 2021.W-0016.001, 2021.W-0016.005, 2021.W-0016.008).

### 2.2 Determination of the optimal dose in AAV-*Slc25a47* mice

Twelve male wild-type C57BL/6J mice were randomly divided into 4 groups containing 3 mice each. Mice were injected via the tail vein with AAV-*Slc25a47* dissolved in 100 μL PBS at the following titers: 0, 0.25∗10^11^, 1.0∗10^11^, 2.5∗10^11^, and 6∗10^11^. Eight weeks after injection, mice were euthanized via cervical dislocation.

### 2.3 Fasting-refeeding in AAV-*Slc25a47* mice

Forty-eight male wild-type C57BL/6J mice were randomly divided into 2 groups with 24 mice each. At 9–12 weeks of age, 24 mice were injected via the tail vein with AAV-*Slc25a47* dissolved in 100 μL PBS at a titer of 2.5∗10^11^. Another group of 24 mice was injected with an equivalent amount of AAV-*Gfp*. Three weeks after injection, half of the mice in each group (N = 12) were fasted for 24 h. The other half of the mice (N = 12) was fasted for 18 h, after which the food was returned for 6 h followed by blood collection and euthanasia. Blood was collected via orbital puncture under isoflurane anaesthesia, followed by euthanasia via cervical dislocation.

### 2.4 High-fat diet in AAV-*Slc25a47* mice

Twenty-four male wild-type C57BL/6J mice were randomly divided into 2 groups with 12 mice each. At 9–12 weeks of age, 12 mice were injected via the tail vein with AAV-*Slc25a47* dissolved in 100 uL PBS at a titer of 2.5∗10^11^. Another group of 12 mice was injected with an equivalent amount of AAV-*Gfp*. Two weeks after injection, the mice were placed on HFD containing 60 energy percent fat (D12492, Research Diets, Inc, New Brunswick) for 11 weeks. During the entire study, the mice were individually housed. Ten weeks after injection, blood was collected via orbital puncture under isoflurane anaesthesia, followed by euthanasia via cervical dislocation.

### 2.5 Indirect calorimetry in *Slc25a47*^−/−^ mice

For this experiment, the mice were on a mixed C57BL/6N and C57BL/6J background. For the indirect calorimetry measurements, male wild-type and *Slc25a47*^*−/−*^ (Tm1b) littermates of 13–16 weeks of age (N = 12/group) were individually housed and placed on a low-fat diet (LFD) containing 10 energy percent fat (D12450, Research Diets, Inc.) for 2 weeks. After 1.5 weeks of LFD, indirect calorimetry measurements were performed to assess energy expenditure, respiratory exchange ratio, activity, and food intake. At baseline, fat mass and lean mass were measured using EchoMRI 100 V (EchoMedical Systems, Houston, TX, USA). Mice were subsequently housed in the indirect calorimetric system with bedding, nest material, and cage enrichment. After an initial acclimatization period of 24 h with ad libitum access to the LFD and water, mice were subsequently monitored for 48 h in the fed and fasted state. For the first 24 h, mice were monitored while having ad libitum access to LFD and drinking water. For the following 24 h, mice were fasted with only access to drinking water. At the end of the measurement, body weight and body composition were again measured using EchoMRI. Since no significant differences were observed for body weight and lean mass between groups, and in 24 h EE between groups (as revealed by non-significant ANCOVA analysis of 24 h EE versus lean mass [22]), EE (kcal/h) is expressed without normalization.

### 2.6 High-fat diet in *Slc25a47*^−/−^ mice

For this experiment, the mice were on a mixed C57BL/6N and C57BL/6J background. Twenty-four male wild-type and twenty-four male *Slc25a47*^*−/−*^ (Tm1b) mice at 15–18 weeks of age were randomly assigned to LFD containing 10 energy percent fat (D12450, Research Diets, Inc.) or a high-fat diet (HFD) containing 45 energy percent fat (D12451, Research Diets, Inc.) for 20 weeks (N = 12/group). The wild-type and *Slc25a47*^*−/−*^ mice were littermates and obtained via heterozygous breeding. Body weight and food intake were assessed weekly. One mouse in the wild-type HFD group was removed from the analysis due to an ambiguous genotype. One mouse in the wild-type HFD group became ill and had to be removed from the study. Two mice were removed from the *Slc25a47*^−/−^ HFD group because they were heterozygous according to re-genotyping and assessment of *Slc25a47* gene expression. One mouse in the *Slc25a47*^−/−^ HFD group became ill and had to be removed from the study.

Three weeks before euthanasia, wild-type and *Slc25a47*^−/−^ mice fed the HFD diet were subjected to an intraperitoneal glucose tolerance test. After a 5-hour fast, the mice were injected intraperitoneally with glucose (1 g/kg body weight) (Baxter, Deerfield, IL). Blood samples from tail vein bleeding were taken and glucose levels at different time points after glucose injection were measured using a GLUCOFIX Tech glucometer and glucose sensor test strips (Menarini Diagnostics, Valkenswaard, The Netherlands). Blood glucose values for 2 mice per group could not be used because they exceeded the maximal detection level.

After 20 weeks of LFD or HFD, blood was collected via orbital puncture under isoflurane anaesthesia, followed by euthanasia via cervical dislocation.

### 2.7 Fasting *Slc25a47*^−/−^ mice, males only

For this experiment, the mice were on a mixed C57BL/6N and C57BL/6J background. Twenty-eight male wild-type and twenty-eight male *Slc25a47*^*−/−*^ (Tm1a and Tm1b) mice at 16–20 weeks of age were randomly assigned to the fed or fasted groups with 14 mice per group. The wild-type and *Slc25a47*^*−/−*^ mice were not littermates and were obtained via parallel breeding. The mice were either fasted for 24 h or remained in the ab libitum fed state. Blood was collected via orbital puncture under isoflurane anaesthesia, followed by euthanasia via cervical dislocation.

### 2.8 Fasting *Slc25a47*^−/−^ mice, males and females

For this experiment, the mice were on a pure C57BL/6J background. Twenty-seven male wild-type and twenty-six male *Slc25a47*^*−/−*^ (Tm1a and Tm1b) mice and twenty female wild-type and twenty-three female *Slc25a47*^*−/−*^ (Tm1a and Tm1b) mice at 11–15 weeks of age were randomly assigned to fed or fasted groups with 10–14 mice per group. The wild-type and *Slc25a47*^*−/−*^ mice were littermates and were obtained via heterozygous breeding. The mice were either fasted for 24 h or remained in the ab libitum fed state. Blood was collected via orbital puncture under isoflurane anaesthesia, followed by euthanasia via cervical dislocation.

### 2.9 High-fat diets differing in fat composition in *Slc25a47*^−/−^ mice

For this experiment, the mice were on a mixed C57BL/6N and C57BL/6J background. Thirty male wild-type and thirty male *Slc25a47*^*−/−*^ (Tm1a and Tm1b) mice at 22–28 weeks of age were randomly assigned to HFD containing 45 energy percent fat (D12451, Research Diets, Inc.) composed of either medium-chain triglycerides oil, milk fat, or mustard oil for 20 weeks (N = 10/group). The overall composition of the diets is shown in Table S1 and the fatty acid composition is shown in Table S2. The wild-type and *Slc25a47*^*−/−*^ mice were not littermates and were obtained via parallel breeding. Blood was collected via orbital puncture under isoflurane anaesthesia, followed by euthanasia via cervical dislocation.

### 2.10 VLDL secretion

For this experiment, the mice were on a mixed C57BL/6N and C57BL/6J background. Fourteen male wild-type and fourteen male *Slc25a47*^*−/−*^ mice at the age of 12–16 weeks were used. The wild-type and *Slc25a47*^*−/−*^ mice were not littermates and were obtained via parallel breeding. Both groups of mice were fasted for 16 h, after which mice were injected intravenously with tyloxapol (500 mg/kg body weight; 15% solution in PBS) (Sigma–Aldrich). Immediately after injection, blood was collected by tail tip incision using haematocrit capillaries containing sodium heparin for the measurement of baseline plasma triglyceride levels. Blood drawing was repeated at 30, 60, 90, 120 and 150 min. Mice were euthanized after the last blood drawing. Blood was spun down for 15 min at 5000 rpm and 4 °C, after which plasma triglyceride levels were measured using a commercially available kit according to the manufacturer's instructions (Liquicolor Mono, Human GmbH, Wiesbaden, Germany).

### 2.11 Quantification of plasma parameters

Blood samples were collected into EDTA-coated tubes (Sarstedt, Nümbrecht, Germany) and centrifuged at 4 °C for 15 min at 12,000*g*. Plasma was collected and stored at −80 °C. Plasma concentrations of glucose (Glucose GOD FS 10′, DiaSys Diagnostic Systems GmbH, Holzheim, Germany), triglycerides (TG) (Liquicolor Mono), cholesterol (cholesterol FS assay, DiaSys Diagnostic Systems GmbH), free fatty acids (Wako Chemicals, Neuss, Germany; HR(2) Kit; Instruchemie), glycerol, β-hydroxybutyrate, acetoacetate, glutamine (Sigma–Aldrich, Houten, the Netherlands) and urea (Nitrogen (BUN) Colorimetric Detection Kit, Thermo Fisher Scientific, Waltham, USA) were determined according to manufacturers’ instructions.

### 2.12 Liver triglycerides

Liver pieces of ∼50 mg were homogenized to a 5% lysate (m/v) in 10 mM Tris, 2 mM EDTA, 0.25 M sucrose, pH 7.5. Homogenates were assayed for triglycerides using a kit for triglycerides (Instruchemie, Delfzijl, the Netherlands).

### 2.13 Liver glycogen

Liver pieces of ∼100 mg were dissolved in 1 M NaOH (10% homogenate) by incubation at 55 °C for 1 h. Next, 1 M HCL in a 1:1 ratio was added, and samples were centrifuged for 5 min at 3000 rpm. Amyloglucosidase (1000 U/mL in 0.2 M acetate buffer; Sigma–Aldrich) was subsequently added to the samples to convert glycogen to glucose, and samples were incubated at 42 °C for 2 h while shaking (700 rpm). Glucose levels were subsequently measured using a commercially available kit.

### 2.14 Histology

Staining of neutral lipids was performed on frozen liver sections using Oil Red O according to standard protocols.

### 2.15 RNA isolation and quantitative PCR

Total RNA was isolated using TRIzol reagent (Life Technologies, Bleiswijk, The Netherlands). For the isolation of total RNA from the liver, the RNeasy mini kit (Qiagen, Venlo, The Netherlands) was used. Next, 500 ng RNA was used as input to synthesize cDNA by using iScript cDNA synthesis kit (Bio-rad Laboratories, Veenendaal, The Netherlands). Gene expression was measured by Sensimix (Bioline, GC Biotech, Alphen aan den Rijn, The Netherlands) on a CFX384 real-time PCR detection system (Bio-Rad Laboratories, Veenendaal, the Netherlands). Expression of *36b4* was used for normalization of data from mouse liver. A list with primer sequences can be found in Table 1.

**Table 1:** List with primers for qPCR.

Primer name	Forward	Reverse
*m36b4*	ATGGGTACAAGCGCGTCCTG	GCCTTGACCTTTTCAGTAAG
*mSlc25a47* (exon 5)	GCCACTGCACTGTTTAGTCAC	ACTCGCAGAGCATAGCATAGG
*mSlc25a47* (exon 3)	ACGGAGGCCAAATACGCAG	TTGACGATACGTGTCCCGGATG
*rSlc25a47*	TGGACGTTATCAAGTCCCGC	GCCTCAGCACAGCCTCATAA
*hSLC25A47*	AGACGGAGCCAAAGTACACAG	AAGACACGGAAGATACCAGGG
*mPparα*	TATTCGGCTGAAGCTGGTGTAC	CTGGCATTTGTTCCGGTTCT
*mSlc25a20*	CCGAAACCCATCAGTCCGTTTAA	ACATAGGTGGCTGTCCAGACAA
*mAcadl*	GAGAAGTGAGTAGAGAGGTCTGG	AACTGCTGTTGAGAGCAAGTC
*mTfam*	ATTCCGAAGTGTTTTTCCAGCA	TCTGAAAGTTTTGCATCTGGGT
*mCpt2*	ACCCTGCCAGAAGTGACAC	ACGAGTTGAATTGAAAAGCCGAA
*mCol1a1*	TGTGTGCGATGACGTGCAAT	GGGTCCCTCGACTCCTACA
*mTimp1*	GCAACTCGGACCTGGTCATAA	CGGCCCGTGATGAGAAACT
*mFgf21*	GTGTCAAAGCCTCTAGGTTTCTT	GGTACACATTGTAACCGTCCTC
*mLonp1*	ATGACCGTCCCGGATGTGT	CCTCCACGATCTTGATAAAGCG
*mHspa9*	AATGAGAGCGCTCCTTGCTG	CTGTTCCCCAGTGCCAGAAC
*mCd68*	CCAATTCAGGGTGGAAGAAA	CTCGGGCTCTGATGTAGGTC
*mAdgre1*	CTTTGGCTATGGGCTTCCAGTC	GCAAGGAGGACAGAGTTTATCGTG

### 2.16 Microarray

Isolated total RNA from livers of mice infected with AAV-*Slc25a47* or AAV-*Gfp* and fed a high-fat diet was used for transcriptome analysis via microarray. RNA integrity was determined using an Agilent 2100 Bioanalyzer with RNA 6000 microchips (Agilent Technologies, Santa Clara, CA). Purified RNA (100 ng) was labeled with the Ambion WT expression kit (Invitrogen) and hybridized to an Affymetrix Mouse Gene 2.1 ST array plate (Affymetrix, Santa Clara, CA). Hybridization, washing, and scanning were carried out on an Affymetrix GeneTitan platform, and readouts were processed and analyzed according to the manufacturer's instructions. Further analysis of the transcriptome data was carried out as described previously [1].

### 2.17 RNA sequencing

Isolated total RNA from livers of 24 h fasted wild-type and *Slc25a47*^−/−^ mice was used for transcriptome analysis via RNA sequencing. RNA integrity was determined using an Agilent 2100 Bioanalyzer with RNA 6000 microchips (Agilent Technologies, Santa Clara, CA). Library construction and subsequent RNA sequencing runs on the BGISEQ-500 platform were conducted at Beijing Genomics Institute (BGI, Hong Kong) as previously described [23].

### 2.18 Targeted metabolomics

Targeted metabolomics was performed on plasma, liver, and liver mitochondria of fasted wild-type and *Slc25a47*^−/−^ mice and on livers of wild-type and *Slc25a47*^−/−^ mice fed high-fat diets differing in fat composition. Metabolomics was executed by Beijing Genomics Institute (BGI). In short, for sample preparation, the appropriate amount of sample and QC was taken (25 μL of plasma and mitochondrial suspension, 25 mg of liver tissue) followed by the addition of 140 μL of 50% water/methanol solution. Next, the derivatization reaction was performed on the sample, QC, and prepared standards. Samples were centrifuged at 12,000r/min, 4 °C for 10 min, after which supernatant was taken for LC-MS/MS analysis. Metabolites were separated and detected by Liquid chromatography-tandem mass spectrometry (LC-MS QTRAP 6500+ (SCIEX, USA) with multiple reaction monitoring (MRM) scanning mode. The BEH C18 (2.1 mm × 10 cm, 1.7 μm) was used as chromatographic column, and for mass spectrometry electron spray ionization (ESI)+/ESI− was used as ion source. For the plasma, liver, and liver mitochondria samples of fasted wild-type and *Slc25a47*^−/−^ mice, a targeted panel containing 350 (HM350) metabolites was used. For the liver samples of wild-type and *Slc25a47*−/− mice fed high-fat diets differing in fat composition, a targeted panel containing 700 (HM700) metabolites was used. Panels were used to quantify various types of amino acids, fatty acids, sugars, bile acids, acylcarnitines, phenol or derivates and indoles.

Based on the KEGG database, metabolic pathway enrichment analysis of differential metabolites was carried out. A metabolic pathway with a P-value less than 0.05 was considered significantly enriched, and the bubble plot was drawn for the pathway with significantly enriched differential metabolites.

### 2.19 Plasmid constructs

Plasmid for *pSlc25a47_Egfp N2* was constructed by cloning the full-length mouse *Slc25a47* cDNA into *pEgfp-N2* (Clonetech, Mountain View, California, USA). Briefly, RNA from mouse liver was reverse transcribed with First Strand cDNA synthesis kit (Thermo Fisher Scientific) and amplified with Phusion High fidelity DNA Polymerase (Thermo Fisher Scientific) Fwd_NheI_slc25A47: *GCATGAGCTAGCACCATGGATTTTGTTGCTGGGGCC* and Rev_BamHI_slc25A47: *GCATGAGGATCCTTGTGAGCAGGCTCTGCGTGAG*. The PCR products were cloned into pEGFP-N2 vector using the NheI-HF and BamHI-HF (New England Biolabs Inc.) restriction enzyme sites. Afterwards, MAX Efficiency ® DH5α™ Competent Cells (Invitrogen) were transformed by heat-shock and grown in Luria–Bertani (LB) agar plates with kanamycin (Sigma–Aldrich). The vector was isolated using Qiagen plasmid maxi kit (Qiagen) according to manufacturer instructions.

### 2.20 Transient transfection and stable cell line

For transient transfections the plasmids p*Slc25a47_Egfp N2* and empty p*Egfp-N2* for control were complexed to polyethylenimine (PEI) (Polyscience Inc., PA, USA) 1:3 plasmid:PEI ratio in serum free DMEM (Lonza, Belgium). After 6 h, the transfection medium was changed to DMEM with 10% FCS. For selection of stably-transfected cells, 24 h after transfection medium was changed to selection medium DMEM with 600 ug/mL G418 (Sigma–Aldrich, The Netherlands). After 48 h incubation, cells were diluted and seeded on 96 well plates with selection medium. At 70% confluency, fluorescent positive wells were re-selected, diluted and replated in 96 well plates. Selected colonies were incubated in maintenance medium (DMEM with 10% FCS and 250 μg/mL G418) and gradually grown into larger volumes.

### 2.21 Colocalization analysis using Mitotrack Red FM

Hepa1-6 cells transiently transfected with *Slc25a47_Egfp-N2* or non-transfected Hepa 1–6 cells were cultured in complete DMEM at standard conditions (37 °C, 5% CO_2_, 95% humidified atmosphere). Cells were seeded on rat tail collagen-coated 15μ 8-well glass bottom slide (Ibidi, Martinsried, Germany). After 24 h, cells were washed and stained for 30 min with 50 nM MitoTrack Red FM (Thermo Fisher Scientific). For imaging, the culture medium was replaced with FluoroBrite DMEM (Thermo Fisher Scientific). Cells were imaged *in vivo* on a Leica TCS SP5 X. Images were acquired sequentially. SLC25A47_EGFP-N2 was excited at 488 nm and fluorescence emission was detected in a spectral window of 495–540 nm. MitoTrack Red FM was excited at 581 nm and detected in a spectral window of 602–650 nm. Every cell was only imaged once. Laser power, zoom factor, and acquisition speed were unchanged throughout the experiment.

### 2.22 Mitochondria isolation and Western Blot

Different centrifugation steps were conducted to isolate the mitochondria of cells and liver tissues. Hepa1-6 cells stably transfected with *Slc25a47_Egfp-N2* or *Egfp-N2* were cultured in antibiotic-free medium to ensure an optimal mitochondrial function. The cells were harvested by using trypsin 1% and washed and centrifuged down two times in ice-cold isolation mitochondrial buffer (IMB) (210 mM mannitol, 70 mM sucrose, 1 mM EDTA, 10 mM HEPES. Complete EDTA -Free Protein inhibitor cocktail tablet (Roche, USA) and 2 mg/mL fatty acid-free BSA was added prior to usage, pH 7.5). This was followed by resuspension of the pellet in IMB including 2 mg/mL albumin to bind fatty acids. The cells were homogenized using a glass Dounce homogenizer performing 60 strokes on ice. The homogenate was centrifuged for 5 min at 2350 rpm to get rid of the whole cell lysate (pellet 1). In the next step, the supernatant was centrifuged for 45 min at 10,900 rpm to obtain the cytosolic fraction (supernatant 1) and the isolated mitochondrial fraction (pellet 2). For Western blot, the mitochondrial pellet was resuspended in 1% CHAPS or gently resuspended in storage buffer for ATP analysis. For Western Blot analysis anti-GFP antibody was used to detect SLC25A47- EGFP fused protein.

### 2.23 ATP content assay

The Molecular ProbesTM ATP Determination Kit (A22066) of Invitrogen with some modifications was used to detect the ATP content in isolated mitochondria of Hepa 1–6 cells stably transfected with *Slc25a47_Egfp-N2* or *Egfp-N2*. Prior to assay, cells were cultured on antibiotic-free medium for 2 passages. The substrates L-(−)-Malic acid (1M) and Sodium Pyruvate (1M) were added in a concentration of 1 μL substrate/1 mL reaction solution. The reaction solution was gently mixed, protected from light and preheated for at least 15 min at the optimal reaction temperature of 28 °C. Luminometric absorption of the mitochondrial samples was measured in a dark 96-well Costar well plate (3915, Corning Incorporated) using a Fluoroskan plate reader, integration time 20 ms (Fluoroskan Ascent FLTM, Thermo Fisher Scientific). During the centrifugation step to spin down the mitochondrial pellet (i.e. 45 min. at 10,900 rpm), the ATP standard curve (0 nM–1000 nM) was measured using 10 μL of ATP stock (5 mM) dilutions in storage buffer. The measured values were subtracted from the background luminometric absorption values of the 90 μL reaction solution. As soon as the isolated mitochondrial pellet was retrieved, it was resuspended in storage buffer by gently pipetting up and down to keep the mitochondria intact. Mitochondrial function was measured within 20 min after the last centrifugation step, due to time-dependent degradation of mitochondria. Firstly, the background luminometric absorption of the 90 μL reaction solution was measured. This was followed by ATP content measurements by the addition of 10 μL of the isolated mitochondria dissolved in storage buffer in technical triplicates. The measurement (90 μL reaction solution + 10 μL mitochondrial sample) minus the background value (90 μL reaction solution) minus the mean of the duplicate negative control samples (90 μL reaction solution + 10 μL of the storage buffer) represents the ATP content. Results were normalized to protein content as measured by Micro BCA Protein Assay Kit (Thermo Fisher Scientific).

### 2.24 High-resolution respirometry

Respirometry analyses were performed using high-resolution respirometry (Oroboros Oxygraph-2k; Oroboros Instruments, Innsbruck, Austria). Before the experiments, calibrations were performed according to the manufacturer's recommendations.

### 2.25 Intact Hepa1-6 cells overexpressing *Slc25a47* or *Egfp*

Hepa1-6 were transiently transfected with p*Slc25a47_Egfp-N2* and empty *pEgfp-N2* at least 90% transfection efficiency in T75 flasks. Each experiment was performed 48 h after transfection with 10^6^ cells per condition and thereafter, results were normalized for protein content. Cells were cultured and measured in DMEM +10% FCS. For experiments with fatty acid-loaded cells, 24 h after transfection, Hepa 1–6 cells were fatty acid loaded with 1.2 mM oleate:palmitate (2:1 ratio) coupled to 3% BSA-DMEM and incubated overnight. Respiratory states were assessed using a Coupling Control Protocol [24]. Briefly, Hepa 1–6 cells were added to the OROBOROS chambers. Within the next 5–10 min, O_2_ flux stabilized reflecting the cellular ROUTINE respiration. Cellular ROUTINE respiration is the aerobic metabolic activity under standard culture medium conditions in the physiological coupling state. Next, LEAK respiration was assessed by adding 2.5 μM oligomycin (an ATP-synthase inhibitor). LEAK respiration represents the O_2_ flux that is maintained to compensate for the proton leak caused after ATP-synthase inhibition by oligomycin and thus represents the respiration independent of ADP phosphorylation. Thereafter, maximal ETC (electron transport capacity) was assessed by the stepwise titration of 1 μM CCCP (a proton translocator that facilitates proton transfer across the membrane) until no further increase of O_2_ flux was observed. ETC represents the uncoupler stimulated respiration, thus the electron-transport-system capacity at noncoupled respiration. Finally, ROX (residual oxygen consumption) was determined by adding 0.5 μM rotenone to inhibit Complex-I and 2.5 μM antimycin A to inhibit Complex-III. ROX is the oxygen consumption that remains after inhibition of the electron transfer pathway, which corresponds to the O_2_ consumption not related to electron transfer. Finally, ATP-linked respiration was calculated as the difference between ROUTINE respiration and LEAK respiration.

### 2.26 Freshly isolated permeabilized liver overexpressing *Slc25a47* or *Egfp*

Livers of mice infected with AAV-*Slc25a47* or AAV-*Gfp* were dissected and immediately transported in MiR05 medium (0.5 mM EGTA, 3 mM MgCl_2_ 6H_2_O, 60 mM lactobionic acid, 20 mM taurine, 10 mM KH_2_PO_4_, 20 mM HEPES, 110 mM d-Sucrose, 1 g/L fatty acid-free BSA, pH 7.0). The protocol for liver permeabilization was adapted from Kuznetsov et al. [25]. Briefly, 2 mg of freshly dissected livers were mechanically permeabilized in ice-cold MiR05 medium using two pairs of forceps. The tips of the forceps were inserted in the middle of the sample, and the tissue was repeatedly torn apart in different directions until pieces of loosely connected liver were obtained. Permeabilized liver pieces were briefly rinsed in MiR05 medium chambers. Respiratory states were assessed using a Substrate-uncoupler-inhibitor titration protocol [24]. Shortly after adding the samples to the chambers, 10 mM glutamate and 2 mM malate were injected to assess Complex I-linked LEAK respiration in the absence of ADP. Glutamate and malate activate dehydrogenases which generate, through coupled reaction, nicotinamide adenine dinucleotide (NADH). The generated NADH can subsequently deliver its electrons into Complex I (CI) and go down the electron transport chain for ATP production by ATP-synthase and O_2_ consumption. However, in the absence of ADP, no oxidative phosphorylation can occur, thus the O_2_ flux reflects the CI-linked LEAK respiration. Next, CI-linked OXIDATIVE respiration was assessed by the stepwise titration of 1 mM ADP. Next, 10 μM of cytochrome C was added to evaluate the integrity of the mitochondrial outer membrane. An increase in respiration at this point would signify the outer mitochondrial membrane is damaged. Next, 10 mM succinate was added to assess CI&CII-linked OXIDATIVE respiration, immediately followed by 1 mM ADP to ensure saturated ADP concentration. Next, CI&CII-linked LEAK respiration was assessed by titration of 2.5 μM oligomycin. Afterward, CI&CII-UNCOUPLED respiration was evaluated by the stepwise titration of 0.5 μM CCCP, until no further increase of O_2_ flux was observed. Finally, CI&CII-ROX was determined by adding 0.5 μM rotenone and 2.5 μM antimycin A.

## 3 Results

### 3.1 SLC25A47 is a PPAR**α**-regulated gene in human and mouse hepatocytes

**Figure 1:**
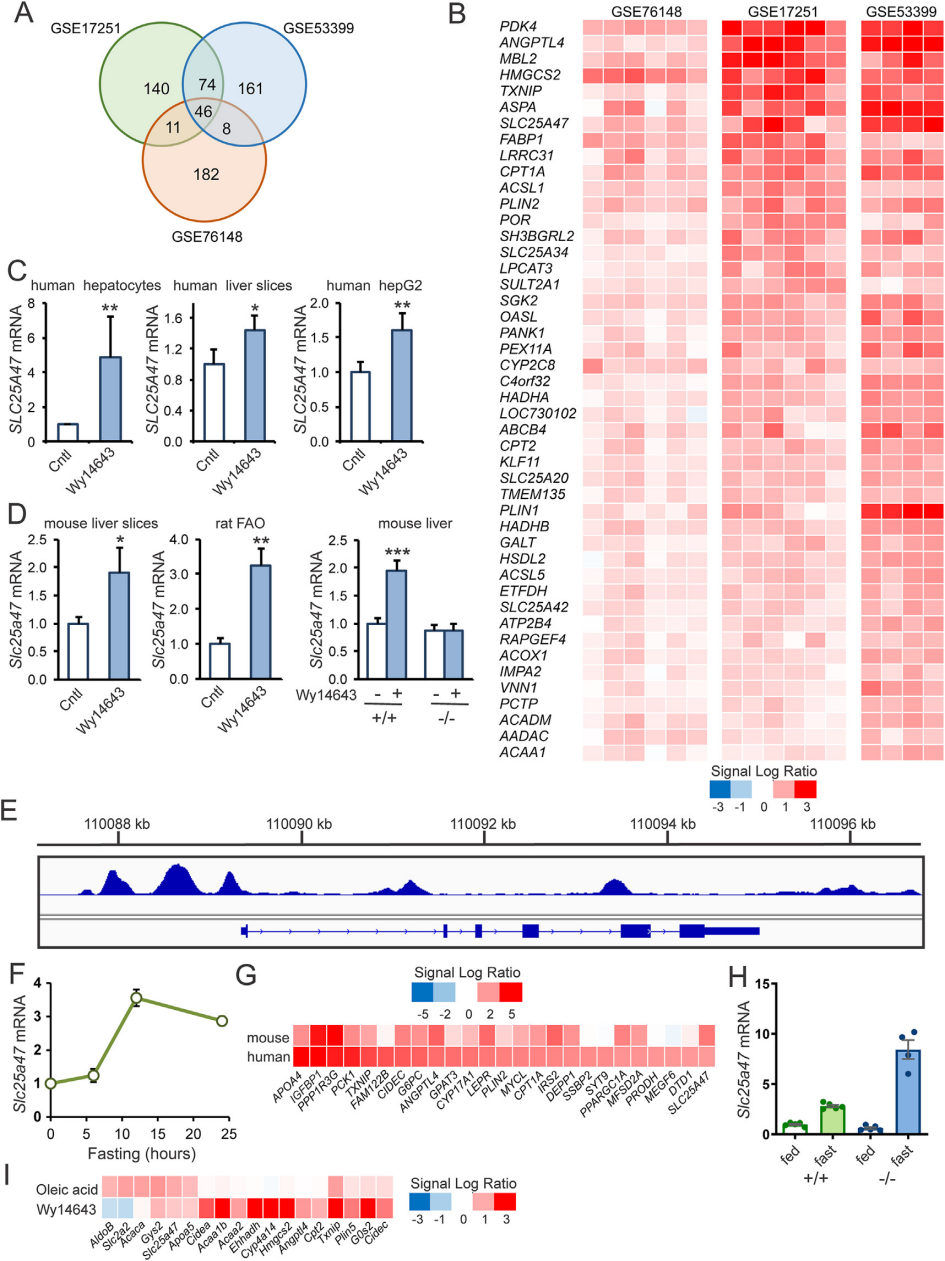
**SLC25A47 is induced by PPARα in human and mouse hepatocytes**. A) Venn diagram showing the overlap in gene regulation in human primary hepatocytes treated with PPARα agonist for 24 h. The following three datasets were included: GSE53399 (GW7647, 1 μM, N = 4) [26], GSE76148 (Wy14643, 50 μM, N = 6) [27], GSE17251 (Wy14643, 50 μM, N = 6) [13]. Genes were considered significantly regulated if *P* < 0.005 and Signal Log Ratio > 0.25 (fold change 1.1892). B) Heatmap showing the expression changes of the 46 genes significantly induced by PPARα activation in all three studies. Each vertical column represents one human donor. C) Effect of Wy14643 on *SLC25A4*7 mRNA in human hepatocytes (24 h, 50 μM), precision cut liver slices (24 h, 50 μM), and HepG2 cells (6 h, 50 μM). D) Effect of Wy14643 on *Slc25a47* mRNA in mouse precision-cut liver slices (24 h, 20 μM), rat liver FAO hepatoma cells (24 h, 5 μM), and mouse liver (5d, 0.1% in food). E) Screenshot of the mouse *Slc25a47* locus showing ChIP-seq profiles of PPARα in mouse liver. F) Effect of fasting on *Slc25a4*7 mRNA in mouse liver. G) Heatmap showing the top 25 genes induced by fasting in human and mouse hepatocytes of hepatocyte humanized mice. Data were extracted from GSE126587 [26]. H) *Slc25a4*7 mRNA in livers of wild-type and PPARα^*−/−*^ mice in the fed or 24 h fasted state. I) Heatmap showing the response in mouse primary hepatocytes of selected genes to Wy14643 (10 μM) and oleic acid (100 μM) treatment for 24 h based on transcriptome analysis. Error bars represent SD for in vitro experiments and SEM for in vivo studies. Asterisk indicates significantly different according to Student's *t*-test. ∗*P* < 0.05, ∗∗*P* < 0.01, ∗∗∗*P* < 0.001.

To identify potential novel PPARα-induced genes in human hepatocytes, we analyzed transcriptome data of three independent and published datasets in which human primary hepatocytes were treated with the PPARα agonist Wy14643 or GW7647 for 24 h [13,26,27]. Genes were considered significantly regulated if P < 0.005 and Signal Log Ratio > 0.25 (fold change 1.1892). A total of 46 genes were significantly induced by PPARα activation in all three studies (Figure 1A). The changes in gene expression of these 46 genes in the hepatocytes from all donors are shown in Figure 1B. Many well-known PPARα target genes are represented in this list, including *CPT1A*, *HMGCS2*, *PDK4*, *ANGPTL4*, *PLIN2,* and *FABP1*. One relatively unknown gene consistently induced by PPARα activation in the three datasets was *SLC25A47*. Expression of *SLC25A47* was specific to the liver in mice and humans (Sup Figure 2A,B). Quantitative PCR confirmed the induction of *SLC25A4*7 mRNA by PPARα activation in human hepatocytes, human liver slices, and human hepatoma HepG2 cells (Figure 1C) [11,13,28]. In addition, *Slc25a47* expression was significantly induced by PPARα activation in mouse hepatocytes, rat FAO hepatoma cells, and mouse liver (Figure 1D) [4,13,29]. Analysis of ChIP-seq data revealed several PPARα binding sites immediately upstream of the transcriptional start site, suggesting that *SLC25A47* is a direct PPARα target gene (Figure 1E) [30].

Since PPARα in the liver is known to be activated by fasting [7,8], we studied the expression of *Slc25a47* in the liver of mice in response to fasting. Expression of *Slc25a47* was indeed induced by fasting, with the highest induction observed after 12 h (Figure 1F). In addition, fasting increased *Slc25a4*7 mRNA levels in hepatocyte-humanized mice in both the mouse and human hepatocytes (Figure 1G). Of all the members of the SLC25 family, *Slc25a47* showed the highest absolute expression and induction by fasting in mouse liver (Sup Figure 2C). In addition, in hepatocyte-humanized mice, where the mouse liver is reconstituted with human hepatocytes yet still containing substantial amounts of mouse hepatocytes, *Slc25a47* was the most highly induced gene by fasting among all SLC25 family members in both mouse and human hepatocytes (Sup Figure 2D). Unlike classical PPARα target genes such as *Ehhadh*, *Cpt2*, and *Hmgcs2* [31], the induction of *Slc25a4*7 by fasting was enhanced rather than suppressed in PPARα^−/−^ mice (Figure 1H).

Also, unlike the classical PPARα targets *Ehhadh*, *Cpt2*, and *Hmgcs2* and resembling the expression of PPARα targets *Apoa5* and *Gys2*, *Slc25a47* expression in primary mouse hepatocytes was induced more strongly by oleic acid than by Wy14643 (Figure 1I). Interestingly, along with many other PPARα target genes [32], hepatic expression of *Slc25a47* was induced in the liver following β3-adrenergic activation (data not shown), as revealed by a publicly available dataset (GSE165699) [33].

### 3.2 SLC25A47 does not function as a mitochondrial uncoupling protein

**Figure 2:**
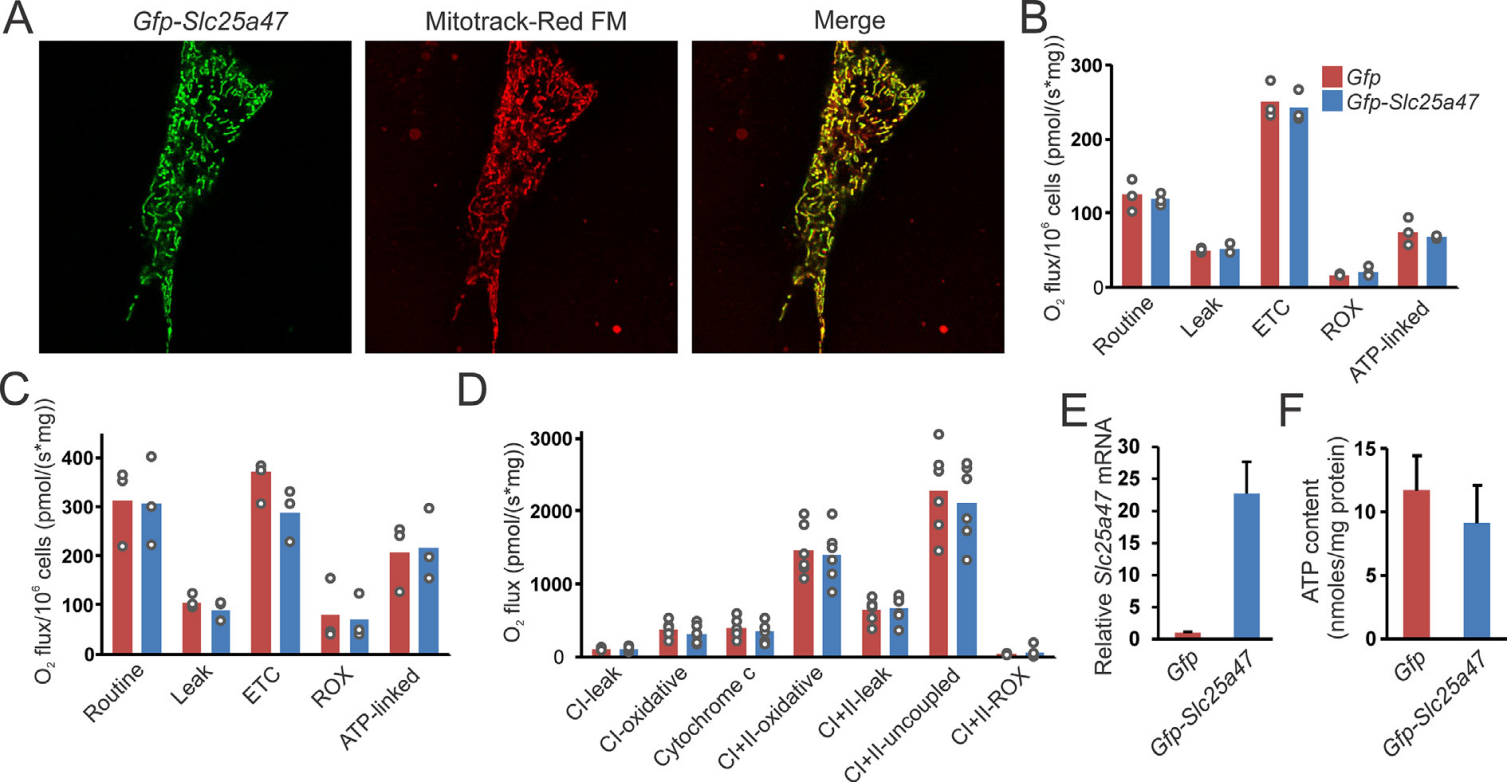
**Effect of SLC25A47 on mitochondrial respiration**. A) Hepa1-6 cells transiently transfected with p*Slc25a47_Egfp-N2* were incubated with MitoTracker™ Red FM and imaged lived on a Leica TCS SP5 X. B) O_2_ flux analysis in Hepa1-6 cells transiently transfected with p*Slc25a47_Egfp-N2* or p*Egfp-N2* (N = 3 measurements per condition). C) O_2_ flux analysis in Hepa1-6 cells transiently transfected with p*Slc25a47_Egfp-N2* or p*Egfp-N2* loaded with fatty acids overnight (1.2 mM oleate:palmitate 2:1) (N = 3 measurements per condition). D) O_2_ flux analysis on permeabilized mouse livers obtained from mice infected with AAV-*Slc25a47* or AAV-*Gfp*. E) *Slc25a47* mRNA expression in Hepa1-6 cells stably transfected with p*Slc25a47_Egfp-N2* or p*Egfp-N2.* F) ATP content in mitochondria isolated from Hepa1-6 cells stably transfected with p*Slc25a47_Egfp-N2* or p*Egfp-N2.* Error bars represent SD.

SLC25A47 was proposed to be a liver-specific mitochondrial uncoupling protein [17,18]. To further investigate the potential role of SLC25A47 as a mitochondrial uncoupler, we performed studies in Hepa 1–6 cells, which do not express *Slc25a47*, nor do any other cancer cell lines part of the Cancer Cell Line Encyclopedia [34]. Consistent with previous literature, with Protein Atlas [17,18], and with the role of SLC25 members as inner mitochondrial membrane transport proteins, we found SLC25A47 to be localized to mitochondria, as indicated by the overlap with the mitochondrial matrix marker Mitotracker Red FM (Figure 2A).

To investigate if SLC25A47 may promote uncoupling, we performed high-resolution respirometry on an Oroboros Oxygraph 2k in Hepa 1–6 cells transiently transfected with *Slc25a47* or *Gfp* (Figure 2B–D). Transfection led to a huge increase in *Slc25a47* expression (Figure 2E). Respiratory states were assessed using a Coupling Control Protocol. Neither under normal conditions (Figure 2B) nor after lipid loading (Figure 2C) did we observe a significant difference between control and *Slc25a47*-expressing cells for any specific respirometry measurements. To further evaluate if SLC25A47 could affect mitochondrial respiration in a different system, we performed respirometry analysis on permeabilized livers of AAV-*Slc25a47* and AAV-*Gfp* infected mice. No significant differences were observed between AAV-*Slc25a47* and AAV-*Gfp* mice for any specific measurements (Figure 2D). Finally, ATP content was not different between Hepa 1–6 cells stably expressing *Slc25a47* or *Gfp* (Figure 2F). Overall, these data do not support an uncoupling role of SLC25A47.

### 3.3 SLC25A47 overexpression minimally impacts whole-body and hepatic metabolic parameters

**Figure 3:**
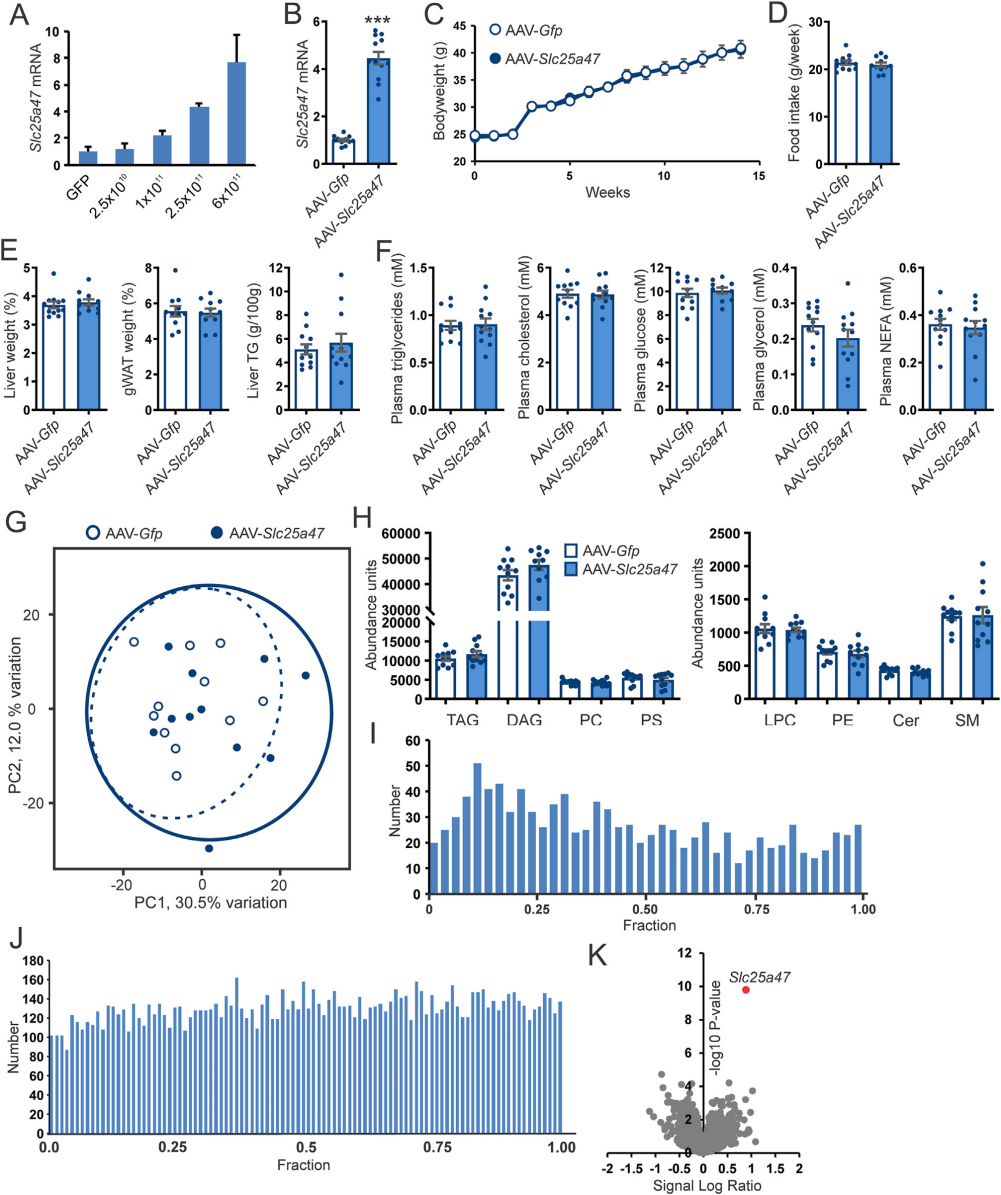
**Effect of hepatic *Slc25a47* overexpression in diet-induced obese mice**. A) *Slc25a4*7 mRNA levels in livers of mice infected with different doses of AAV-*Slc25a47* (N = 2 per group). Error bars are SD. B) Hepatic *Slc25a4*7 mRNA in mice injected with 2.5 × 10^11^ genomic copies of AAV-*Slc25a47* or AAV-*Gfp*. Mice were placed on a HFD 2 weeks later for 8 weeks. N = 12/group. C) Bodyweight. D) Average food intake during the 8 weeks of HFD. E) Tissue weights and liver triglyceride content. F) Plasma metabolites. G) Principal component analysis on liver lipidomics. N = 10/group. H) Hepatic levels of the main lipid species. TAG, triacylglycerol; DAG, diacylglycerol; PC, phosphatidylcholine; PS, phosphatidylserine; LPC, lysophosphatidylcholine; PE, phosphatidylethanolamine; Cer, ceramide; SM, sphingomyelin. I) P-value distribution of the liver lipidomics analysis. J) P-value distribution of the liver transcriptomics analysis. N = 8/group. K) Volcano plot of the liver transcriptome. Error bars represent SEM. Asterisk indicates significantly different from AAV-*Gfp* according to the Student's *t*-test (∗∗∗ = *P* < 0.001).

To study the functional role of SLC25A47 in vivo, we overexpressed *Slc25a47* in mouse liver using adeno-associated virus (AAV). Injection of AAV expressing *Slc25a47* increased hepatic *Slc25a47* expression to a maximum of 7-fold at the highest dose (Figure 3A). Further studies were done using an AAV titer of 2.5 × 10^11^ genomic copies. We first studied the effects of *Slc25a47* overexpression under conditions of obesity and fatty liver. Accordingly, mice were infected with AAV-*Slc25a47* and placed on a high-fat diet (HFD) two weeks later. After 8 weeks of HFD and 10 weeks post-injection, *Slc25a4*7 mRNA levels were more than 4-fold higher in mice injected with AAV expressing *Slc25a47* compared to *Gfp* (Figure 3B). Weight gain (Figure 3C) and food intake (Figure 3D) were not significantly affected by *Slc25a47* overexpression, as were liver and adipose tissue weights and liver triglyceride content (Figure 3E). Plasma triglycerides, non-esterified fatty acids (NEFA), glucose, and cholesterol were not significantly different between AAV-*Slc25a47* and AAV-*Gfp* mice (Figure 3F). To gain further insight into the effect of *Slc25a47* overexpression, we performed lipidomics analysis. No distinct clustering of the two sets of mice was observed (Figure 3G). Also, the levels of the major lipid classes were not significantly different between the two groups (Figure 3H). Not a single lipid met the statistical significance threshold of FDR < 0.05 and the P-value distribution was flat (Figure 3I). To further examine the potential impact of *Slc25a47* overexpression on the liver, we performed transcriptome analysis. As for the lipidomics data, the P-value distribution was flat (Figure 3J) and no distinct clustering of the AAV-*Slc25a47* and AAV-*Gfp* mice was observed (not shown). The only gene that met the statistical significance threshold of FDR < 0.05 was *Slc25a47* (Figure 3K), indicating the minimal effect of *Slc25a47* overexpression on hepatic gene expression.

**Figure 4:**
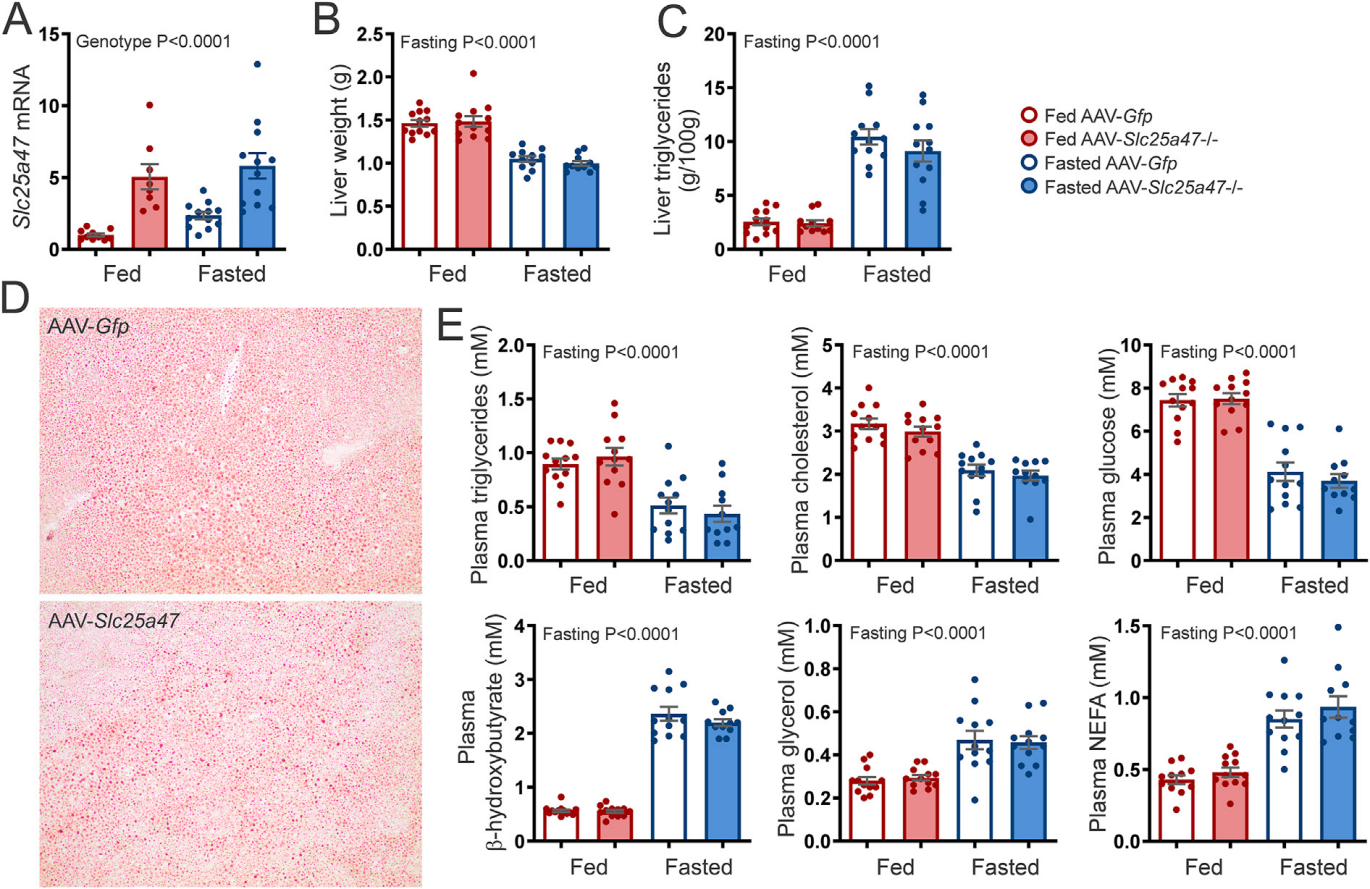
**Effect of hepatic *Slc25a47* overexpression in refed and fasted mice**. A) *Slc25a4*7 mRNA levels in livers of refed and 24 h fasted mice infected with 2.5 × 10^11^ genomic copies of AAV-*Slc25a47* or AAV-*Gfp*. B) Liver weight. C) Liver triglyceride content. D) Oil red O staining. E) Plasma levels of glucose, triglycerides, cholesterol, glycerol, NEFA, and β-hydroxybutyrate. N = 12/group. Data were analyzed by two-way ANOVA. Error bars represent SEM.

Since *Slc25a47* expression is induced by fasting, we next studied the effect of *Slc25a47* overexpression in fed and fasted mice. AAV-mediated *Slc25a47* overexpression significantly increased *Slc25a4*7 mRNA levels in the liver in the fed and fasted state (Figure 4A). No significant effect was observed of AAV-mediated *Slc25a47* overexpression on liver weight (Figure 4B) and hepatic triglyceride levels (Figure 4C). Consistent with this finding, neutral lipid staining of the liver by Oil Red O did not reveal any differences between the AAV-*Slc25a47* and AAV-*Gfp* mice (Figure 4D). Finally, plasma glucose, triglyceride, cholesterol, glycerol, NEFA, and β-hydroxybutyrate levels were not significantly different between AAV-*Slc25a47* and AAV-*Gfp* mice (Figure 4E). Overall, these data do not show an effect of *Slc25a47* overexpression on whole-body and hepatic metabolic parameters in fasted and diet-induced obese mice.

### 3.4 Limited metabolic disturbances in SLC25A47-deficient diet-induced obese mice

**Figure 5:**
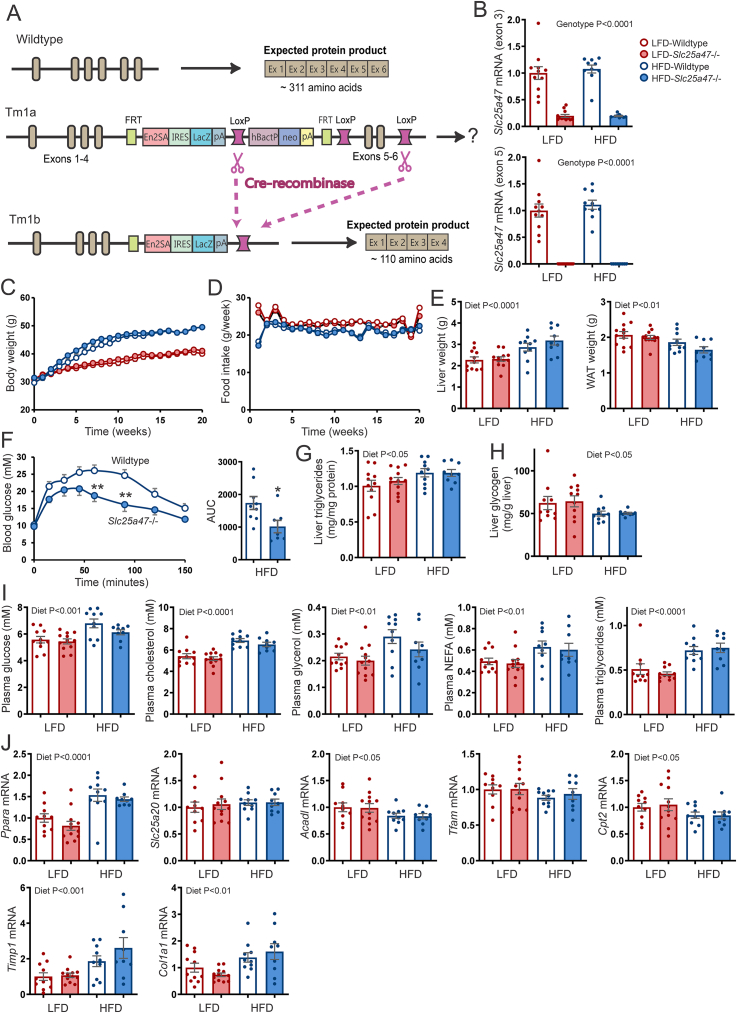
**Effect of hepatic *Slc25a47* ablation on diet-induced obesity**. A) Schematic map of the strategy for inactivation of *Slc25a47*. B) *Slc25a4*7 mRNA in livers of wild-type and *Slc25a47*^−/−^ mice fed LFD (N = 11 wild-type, N = 12 *Slc25a47*^−/−^) or HFD (N = 10 for wild-type, N = 9 for *Slc25a47*^−/−^) for 20 weeks, as determined by qPCR using primers targeting exon 1–3 or exon 5–6. C) Body weights during the course of the low-fat or high-fat feeding. D) Mean food intake per mouse per week. E) Liver and gonadal fat pad (gWAT) weight. F) Intraperitoneal glucose tolerance test (1 g/kg body weight) and area under the curve (AUC). G) Liver triglyceride content. H) Liver glycogen content. I) Plasma levels of glucose, triglycerides, NEFA, glycerol, and cholesterol. J) Hepatic mRNA levels of selected genes involved in lipid metabolism and fibrosis. The wild-type and *Slc25a47*^−/−^ mice were littermates and obtained via heterozygous breeding (mixed C57BL/6J and C57BL/6N background). Data were analyzed by two-way ANOVA or Student's *t*-test (∗*P* < 0.05, ∗∗*P* < 0.01). Error bars represent SEM.

A possible reason why overexpression of *Slc25a47* did not elicit any metabolic phenotype may be that *Slc25a47* is already expressed at high levels in the liver. Accordingly, we further studied the effect of inactivation of *Slc25a47*. To that end, we acquired *Slc25a47*-mutant mice from the EUCOMM/KOMP repository, which was generated by the knockout-first strategy [35]. Mice heterozygous for the Tm1a allele were cross-bred with transgenic mice in which Cre recombinase was expressed in hepatocytes under the control of the Albumin gene promoter (Albumin-Cre), leading to the generation of homozygous Tm1b mice (Figure 5A). The Tm1b mice lack exons 5 and 6, as verified by qPCR (Figure 5B), giving in theory rise to a truncated *Slc25a4*7 mRNA. Levels of exon 3- and exon 5-positive *Slc25a4*7 mRNA in *Slc25a47*^−/−^ mice were markedly reduced compared to wild-type mice (Figure 5B). Unfortunately, we were unable to verify the absence of SLC25A47 protein; none of the many commercial antibodies tested detected SLC25A47 in wild-type mouse liver and Hepa1-6 and HepG2 cells overexpressing *Slc25a47*.

To examine the possible metabolic consequences of SLC25A47 deficiency, we placed the wild-type and *Slc25a47*^−/−^ mice on a HFD for 20 weeks to induce obesity and insulin resistance. Another cohort of wild-type and *Slc25a47*^−/−^ mice were fed a LFD for the same duration. No significant difference in body weight gain (Figure 5C) and food intake (Figure 5D) was observed between wild-type and *Slc25a47*^−/−^ mice, either on the LFD or HFD. Also, no significant differences in liver and gonadal fat pad (gWAT) weight were observed between these two sets of mice (Figure 5E). By contrast, glucose tolerance was significantly improved in the *Slc25a47*^−/−^ mice compared to the wild-type mice (Figure 5F), supported by a significantly lower area under the curve, while basal (T = 0) levels were similar. No significant differences between wild-type and *Slc25a47*^*−/−*^ mice were observed for hepatic triglyceride (Figure 5G) and glycogen (Figure 5H) levels. Also, no significant differences were observed in plasma glucose, cholesterol, triglycerides, glycerol, and NEFA between the two sets of mice (Figure 5I). Since SLC25A47 was previously positively linked to fatty acid oxidation [36], we measured the hepatic expression of *Ppara* and selected PPARα target genes (Figure 5J). No significant differences in the expression of *Ppara*, *Cpt2*, *Acadl*, *Slc25a20*, and *Tfam* were observed between wild-type and *Slc25a47*^−/−^ mice. In addition, no differences were found in the expression of genes connected with hepatic fibrosis, *Timp1* and *Col1a1* (Figure 5J). Although our analysis was not exhaustive, collectively, our data suggest that SLC25A47 deficiency minimally impacts hepatic metabolism in diet-induced obese mice, except for a modest improvement in glucose tolerance.

### 3.5 Fasted SLC25A47-deficient mice have decreased plasma triglyceride and glycerol levels

**Figure 6:**
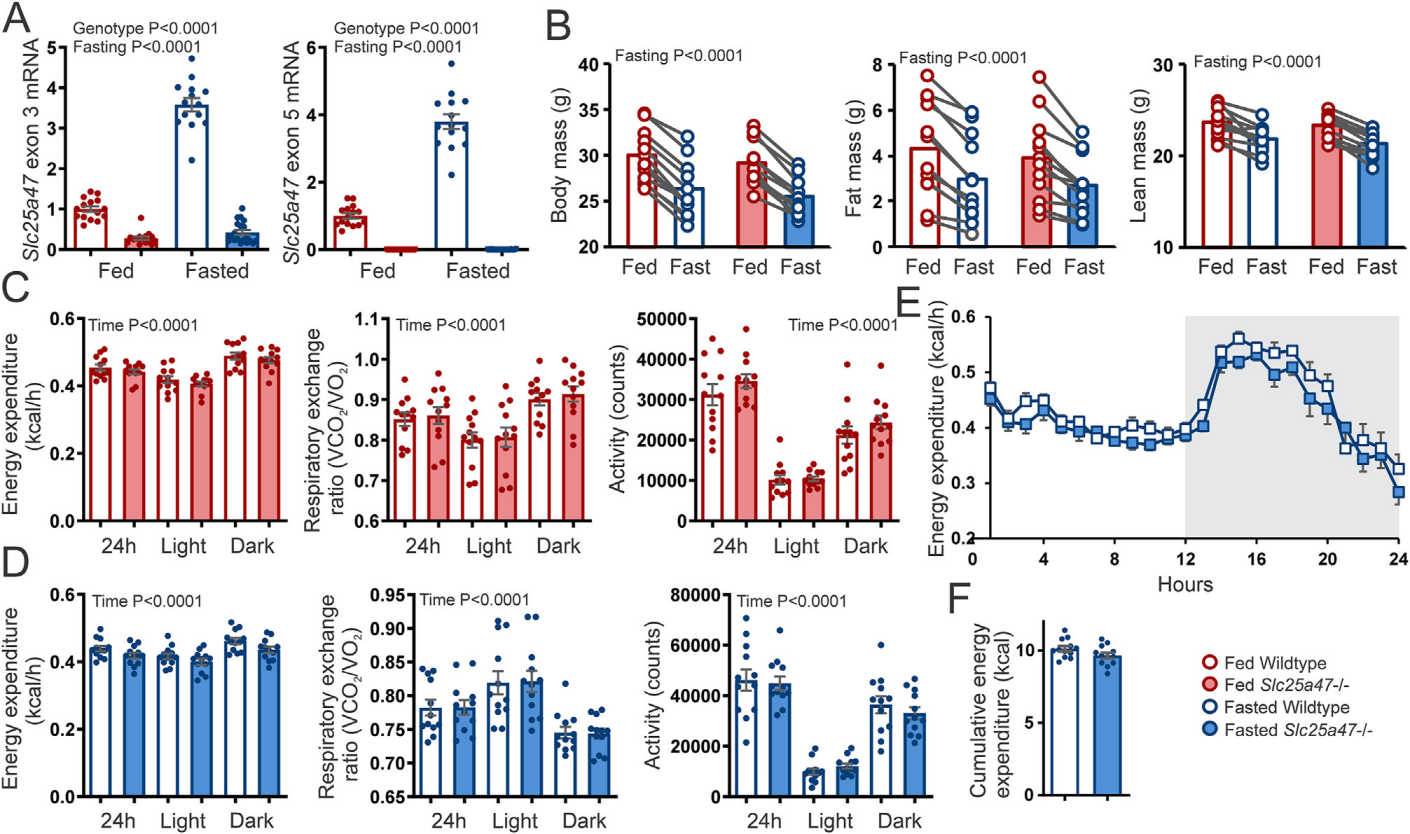
**Effect of hepatic *Slc25a47* ablation on energy metabolism**. A) *Slc25a4*7 mRNA in livers of fed and 24 h fasted wild-type and *Slc25a47*^−/−^ mice, as determined by qPCR using primers targeting exons 1–3 or exons 5–6. B) Body mass, lean mass, and fat mass in wild-type and *Slc25a47*^*−/−*^ mice prior to and after a 24 h fast (N = 12/group). C) Mean energy expenditure, respiratory exchange ratio, and activity level in wild-type and *Slc25a47*^*−/−*^ mice during ad libitum feeding (LFD). D) Mean energy expenditure, respiratory exchange ratio, and activity level in wild-type and *Slc25a47*^*−/−*^ mice during fasting. E) Energy expenditure in wild-type and *Slc25a47*^*−/−*^ mice during 24 h fasting. F) Area Under the Curve for energy expenditure during 24 h fasting. The wild-type and *Slc25a47*^−/−^ mice were littermates and obtained via heterozygous breeding (mixed C57BL/6J and C57BL/6N background). Data were analyzed by two-way ANOVA, two-way repeated measures ANOVA, or Student's *t*-test. Error bars represent SEM.

Since *Slc25a4*7 mRNA levels go up during fasting, the effects of *Slc25a47* ablation may be more pronounced during fasting. Therefore, wild-type and *Slc25a47*^−/−^ mice were fed a normal chow diet and studied with ad libitum access to food (fed state) or fasted for 24 h. Exon 3- and exon 5-positive *Slc25a4*7 mRNA levels were increased by fasting in wild-type mice and were markedly reduced or undetectable in *Slc25a47*^−/−^ mice (Figure 6A). Body mass, fat mass, and lean mass decreased upon fasting but were not significantly different between wild-type and *Slc25a47*^−/−^ mice (Figure 6B). Fed and fasted wild-type and *Slc25a47*^−/−^ mice were next studied by indirect calorimetry. As expected, energy expenditure and activity were higher in the active dark period than in the light period (Figure 6C,D). Concomitantly, the respiratory exchange ratio was higher in the dark period than in the light period in the fed state (Figure 6C), suggesting higher carbohydrate oxidation in concert with higher activity levels in the dark period, whereas the opposite pattern was observed in the fasted state (Figure 6D). Importantly, none of the above parameters were significantly different between wild-type and *Slc25a47*^−/−^ mice, either in the fed or fasted state (Figure 6C,D). The mean energy expenditure during the course of fasting was slightly lower in the *Slc25a47*^−/−^ mice than in the wild-type mice. However, this difference was not statistically significant (Figure 6E–F). Our data thus indicate that SLC25A47 deficiency does not influence whole-body energy expenditure.

**Figure 7:**
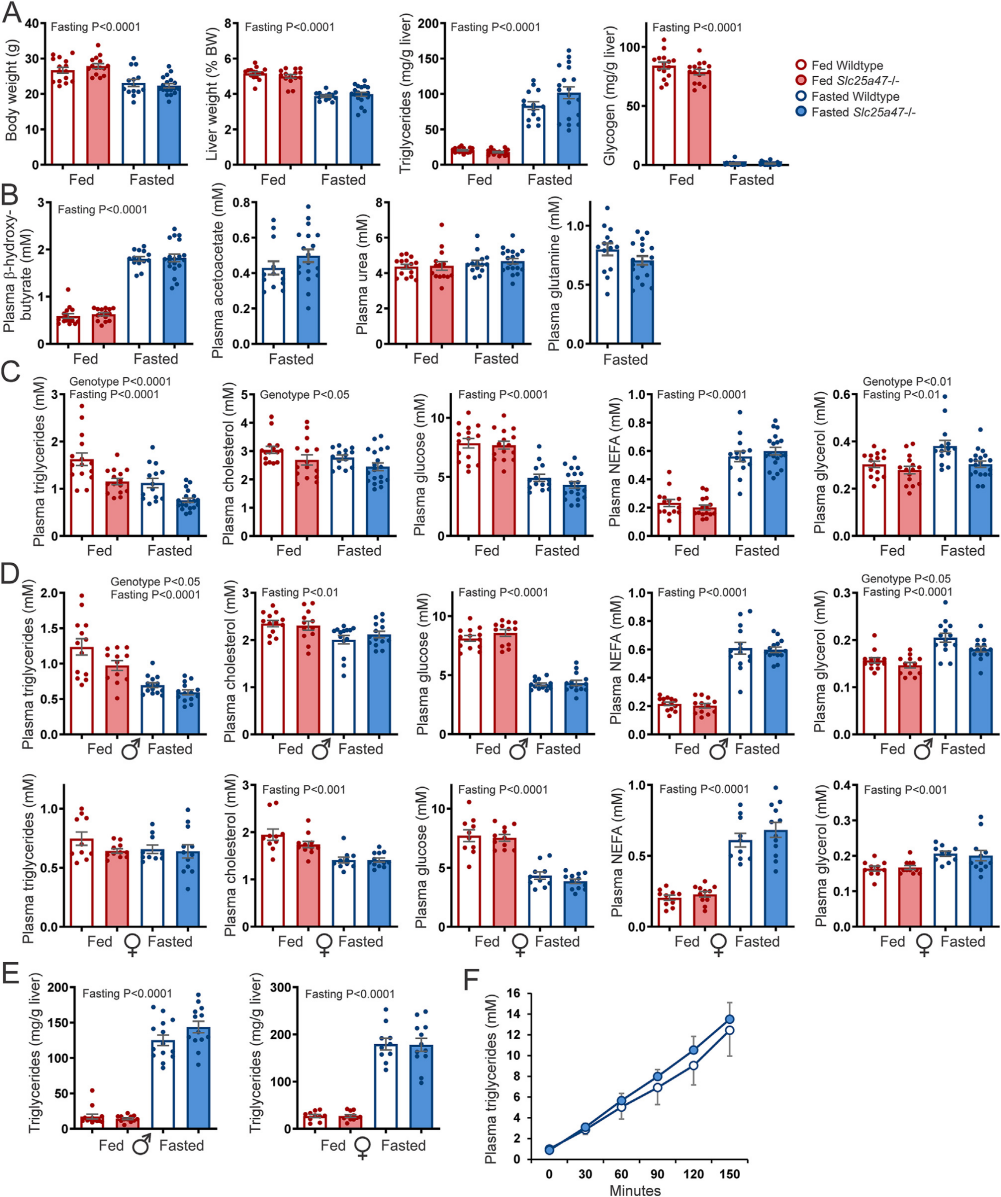
**Effect of hepatic *Slc25a47* ablation in fed and 24 h fasted mice**. A) Bodyweight, liver weight, liver triglyceride content, and liver glycogen content in fed and 24 h fasted wild-type and *Slc25a47*^−/−^ mice (N = 14–19/group). B) Plasma levels of β-hydroxybutyrate, acetoacetate, urea, and glutamine in fed and 24 h fasted wild-type and *Slc25a47*^−/−^ mice (N = 14–19/group). C) Plasma levels of triglycerides, cholesterol, glucose, NEFA, and glycerol in fed and 24 h fasted wild-type and *Slc25a47*^−/−^ mice (N = 14–19/group). D) Plasma levels of triglycerides, cholesterol, glucose, NEFA, and glycerol in a different cohort of male and female fed and 24 h fasted wild-type and *Slc25a47*^−/−^ mice (N = 12–14/group for males, N = 10–12/group for females). E) Liver triglyceride content. F) VLDL production test (N = 14/group). Mice were fasted for 16 h, followed by intravenous injection of tyloxapol (500 mg/kg body weight; 15% in PBS) and subsequent blood drawings via tail tip incision. The wild-type and *Slc25a47*^−/−^ mice were littermates and obtained via heterozygous breeding (D,E; pure C57BL/6J background) or were not littermates and obtained via parallel breeding (A,B,C,F; mixed C57BL/6J and C57BL/6N background). Data were analyzed by two-way ANOVA or Student's *t*-test. Error bars represent SEM.

We further studied the potential impact of SLC25A47 deficiency on hepatic and whole-body metabolic parameters. Body weight, liver weight, and liver glycogen content were significantly lower in the fasted compared to fed mice, while liver triglyceride content was higher in the fasted state (Figure 7A). However, no significant differences were observed between wild-type and *Slc25a47*^−/−^ mice. Next, we measured the plasma levels of various metabolites for which no clear mitochondrial transporter has been identified so far, including β-hydroxybutyrate, acetoacetate, urea, and glutamine. Again, no significant differences were observed between wild-type and *Slc25a47*^−/−^ mice (Figure 7B). Intriguingly, plasma glycerol, cholesterol, and triglyceride levels were significantly reduced in *Slc25a47*^−/−^ mice compared to wild-type mice (Figure 7C). By contrast, plasma glucose and NEFA levels were unaltered in *Slc25a47*^−/−^ mice (Figure 7C).

To validate the reduction in these specific plasma metabolites, we performed a new study in fed and fasted wild-type and *Slc25a47*^−/−^ mice after 5 additional rounds of backcrossing on a C57BL/6J background. Additionally, we now included both male and female mice. In the male but not female mice, plasma glycerol and triglyceride levels were again significantly lower in *Slc25a47*^−/−^ mice compared to wild-type mice (Figure 7D). By contrast, plasma cholesterol, glucose, and NEFA were not significantly affected by genotype (Figure 7D). These data indicate that SLC25A47 deficiency is associated with lower plasma triglycerides and glycerol in a sex-specific manner. No significant effect of SLC25A47 deficiency on liver triglyceride content (Figure 7E), body weight (Sup Figure 3A), and liver weight (Sup Figure 3B) was observed in fed and fasted male and female mice.

Reductions in plasma triglyceride levels can be caused by altered production/secretion of hepatic triglycerides, or by altered triglyceride uptake by extrahepatic tissues [37]. To test whether hepatic VLDL secretion was affected by SLC25A47, wild-type and *Slc25a47*^−/−^ mice were fasted for 24 h followed by injection of the lipoprotein lipase inhibitor tyloxapol, thus preventing extrahepatic triglyceride uptake. Injection with tyloxapol resulted in a similar increase in plasma triglycerides in wild-type and *Slc25a47*^−/−^ mice (Figure 7F). These data suggest that the decreased plasma triglycerides in *Slc25a47*^−/−^ mice are not caused by decreased VLDL-triglyceride secretion.

### 3.6 Impact of SLC25A47 deficiency at transcriptome and metabolome level

**Figure 8:**
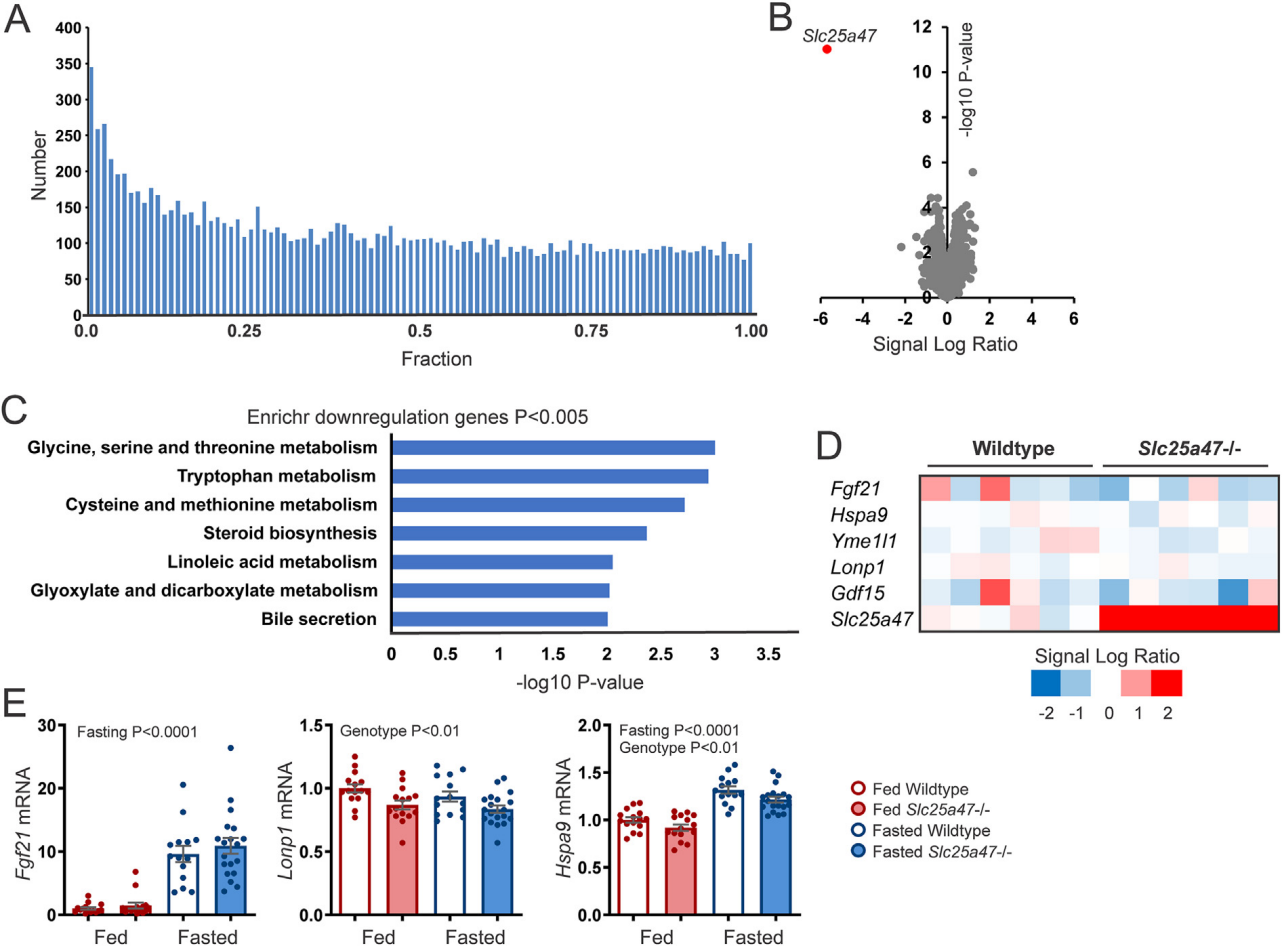
**Effect of hepatic*Slc25a47* ablation in fed and fasted mice onhepaticgene expression**. A) P-value distribution of liver transcriptome analysis comparing 24 h fasted wild-type and *Slc25a47*^−/−^ mice (N = 6/group). B) Volcano plot of the liver transcriptome of 24 h fasted wild-type and *Slc25a47*^*−/−*^ mice. C) EnrichR pathway analysis on the genes downregulated in *Slc25a47*^*−/−*^ mice compared to wild-type mice (*P* < 0.005). D) Heatmap of selected genes involved in the mitochondrial stress response. E) qPCR of selected genes involved in the mitochondrial stress response (N = 14–19/group). The wild-type and *Slc25a47*^−/−^ mice were not littermates and were obtained via parallel breeding (mixed C57BL/6J and C57BL/6N background). Data were analyzed by two-way ANOVA. Error bars represent SEM.

To gain a better understanding of the impact of SLC25A47 deficiency on the liver, we performed RNA sequencing on livers of fasted wild-type and *Slc25a47*^−/−^ mice. The P-value distribution and volcano plot suggested a relatively small effect of Slc25A47 deficiency on the hepatic transcriptome (Figure 8A,B). The only two genes that met the significance threshold of FDR < 0.05 were *Slc25a47* itself and *Nnt* (Nicotinamide Nucleotide Transhydrogenase). The latter gene is mutated and suppressed in C57BL/6J mice and may thus be an artifact of the mixed C57BL/6 genetic background. Pathway analysis by EnrichR on the downregulated genes with P < 0.005 produced various pathways related to amino acid and lipid metabolism (Figure 8C). Unlike a previous study [19], we did not observe any changes in the expression of mitochondrial stress response genes, such as *Fgf21*, *Lonp*, *Hspa9*, and *Yme1l1* (Figure 8D,E).

**Figure 9:**
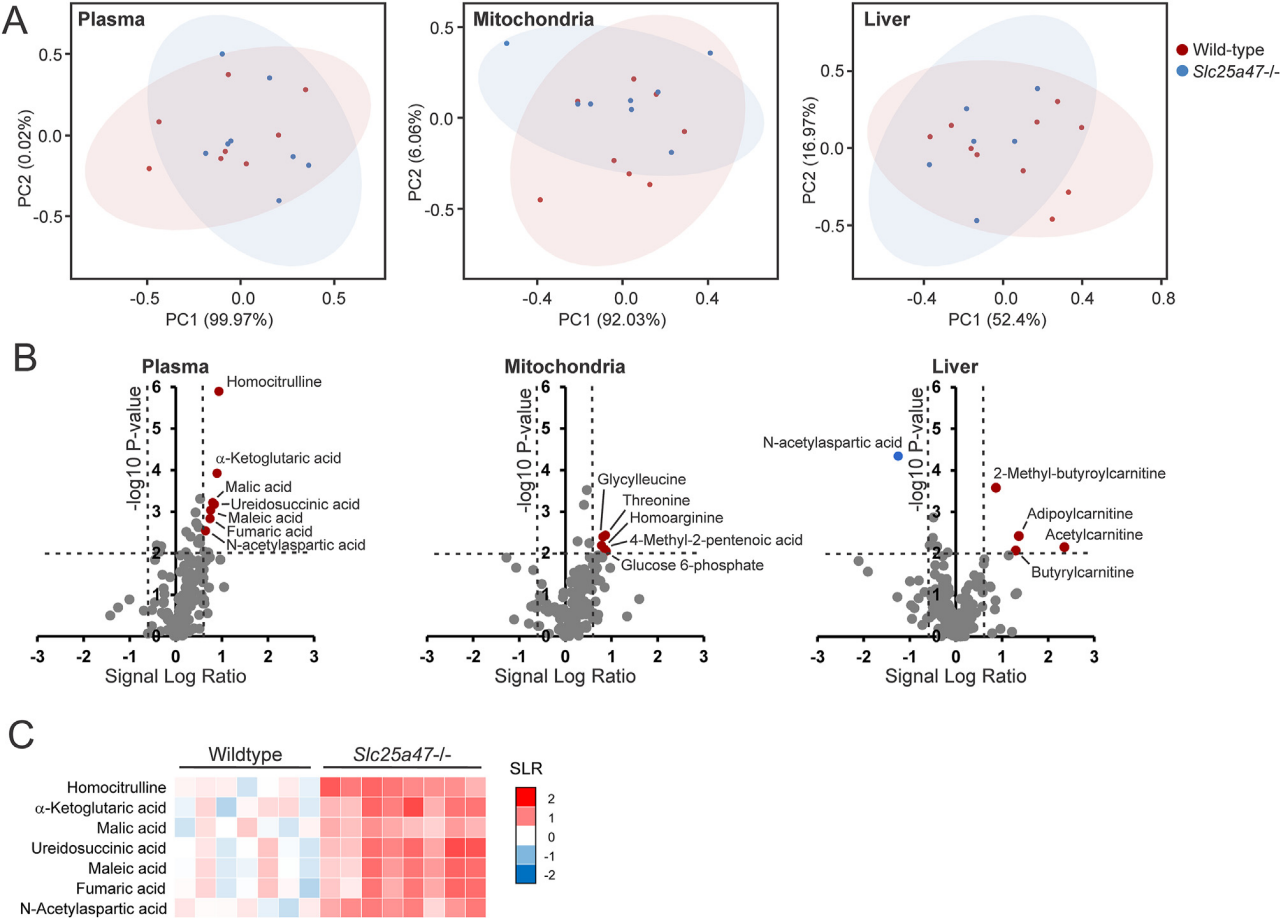
**Effect of hepatic *Slc25a47* ablation in fed and fasted mice on the hepatic metabolome**. A) Principal component analysis (PCA) plot of the metabolome of plasma, liver, or liver mitochondria from fasted wild-type and *Slc25a47*^*−/−*^ mice (N = 8/group). B) Volcano plot of the metabolome of plasma, liver, or liver mitochondria from 24 h fasted wild-type and *Slc25a47*^*−/−*^ mice. Metabolites in color indicate significantly regulated (P < 0.01, fold change > 1.5). C) Heatmap showing significantly upregulated (P < 0.01, fold change > 1.5) metabolites in plasma of fasted *Slc25a47*^*−/−*^ and wild-type mice. The wild-type and *Slc25a47*^−/−^ mice were not littermates and were obtained via parallel breeding (mixed C57BL/6J and C57BL/6N background).

To gain further insights into the impact of SLC25A47 deficiency, we next performed targeted metabolomics on plasma, liver, and liver mitochondria of fasted wild-type and *Slc25a47*^−/−^ mice, using a panel of 350 metabolites consisting of various types of amino acids, carbohydrates, fatty acids, bile acids, and other metabolic intermediates involved in various metabolic pathways such as the TCA cycle, glycolysis, and fatty acid metabolism. Examination of the P-value distribution showed that the largest effects of SLC25A47 deficiency were observed in plasma (Sup Figure 4A). Nonetheless, PCA plots revealed no clear separation between the wild-type and *Slc25a47*^−/−^ mice in all three matrices (Figure 9A). Using a P-value threshold of P < 0.01 (or FDR threshold of 0.05) and absolute fold change of 1.5, plasma levels of homocitrulline, α-ketoglutaric acid, malic acid, ureidosuccinic acid, maleic acid, fumaric acid, and N-acetylaspartic acid were elevated in *Slc25a47*^−/−^ compared to wild-type mice (Figure 9B,C). These metabolites are either TCA cycle intermediates and/or involved in amino acid metabolism. Pathway analysis supported the effect of SLC25A47 deficiency on plasma metabolites involved in amino acid metabolism and the TCA cycle, as well as the oxidation of branched-chain amino acids (Sup Figure 4B). In mitochondria, none of the metabolites met the FDR threshold of 0.05 and fold change of 1.5, although a few metabolites had P < 0.01 (Figure 9B). Similarly, in mitochondria, pathway analysis revealed an effect of SLC25A47 deficiency on metabolites involved in amino acid metabolism (Sup Figure 4B). Finally, pathway analysis in whole liver tissue showed an impact of SLC25A47 deficiency on the oxidation of branched-chain and medium-chain fatty acids, as well as amino acid metabolism (Sup Figure 4B). Only two metabolites met the FDR threshold of 0.05 and fold change > 1.5, which were N-acetylaspartic acid and 2-methyl-butyroylcarnitine. The latter is part of a group of short-chain acyl-carnitines elevated in *Slc25a47*^−/−^ compared to wild-type mice, including adipoylcarnitine, acetylcarnitine, and butyrylcarnitine (all P < 0.01) (Figure 9B). Short-chain acylcarnitines are the products of peroxisomal oxidation of branched and very long-chain fatty acids. They are shuttled to the mitochondria, where they are completely oxidized to CO_2_ and H_2_O [38].

**Figure 10:**
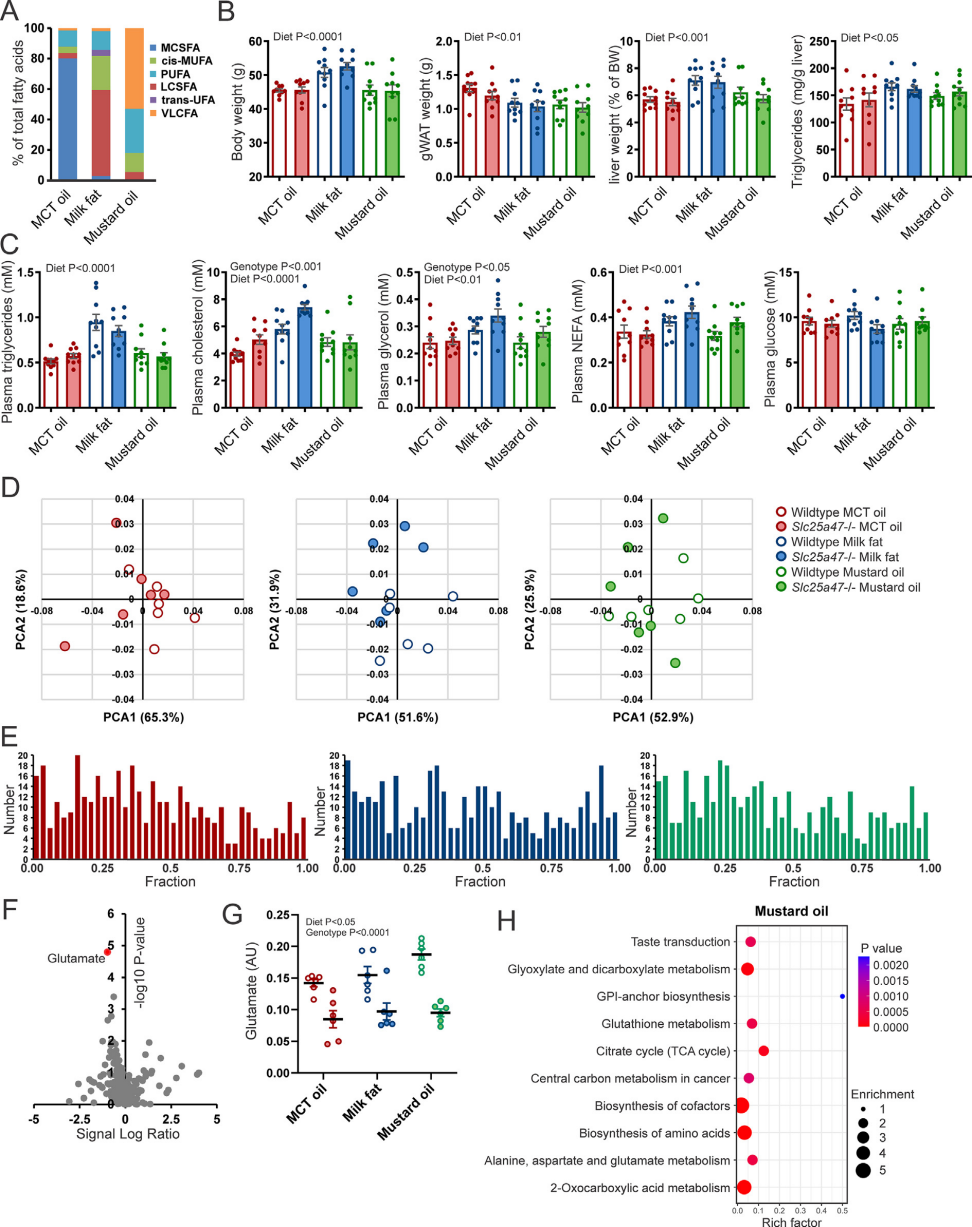
**Effect of hepatic *Slc25a47* ablation in mice fed different high-fat diets**. A) Fatty acid composition of the three test diets provided to wild-type and *Slc25a47*^*−/−*^ mice for 20 weeks. MCSFA, medium-chain saturated fatty acids; cis-MUFA, cis-mono-unsaturated fatty acids; PUFA, poly-unsaturated fatty acids; LCSFA, long-chain saturated fatty acids; trans-UFA, trans-unsaturated fatty acids; VLCFA, very long-chain fatty acids. The individual fatty acids are presented in Table S2. B) Body weight, weight of the gonadal fat pad (gWAT), liver weight, and liver triglyceride content in wild-type and *Slc25a47*^*−/−*^ mice fed HFD composed of medium-chain triglycerides (MCT), milk fat, or mustard oil (N = 10/group). C) Plasma levels of triglycerides, cholesterol, glucose, NEFA, and glycerol. D) Principal component analysis (PCA) of the whole liver metabolome comparing wild-type and *Slc25a47*^*−/−*^ mice on the three test diets. E) P-value distribution of the whole liver metabolome comparing wild-type and *Slc25a47*^*−/−*^ mice on the three test diets. F) Volcano plot of the whole liver metabolome comparing wild-type and *Slc25a47*^*−/−*^ mice fed mustard oil. G) Relative glutamate levels in whole liver based on metabolome analysis. H) Pathway analysis on the whole liver metabolome comparing wild-type and *Slc25a47*^*−/−*^ mice fed mustard oil. The wild-type and *Slc25a47*^*−/−*^ mice were not littermates and were obtained via parallel breeding (mixed C57BL/6J and C57BL/6N background). Data were analyzed by two-way ANOVA. Error bars represent SEM.

Overall, metabolomics did not point towards a single metabolite being strongly disrupted by SLC25A47 deficiency. Rather, the data show an impact of SLC25A47 deficiency on plasma, whole liver, and mitochondrial levels of metabolites involved in amino acid metabolism and the metabolism of various types of fatty acids. Since *Slc25a47* is regulated by PPARα and PPARα is the master regulator of peroxisomal and mitochondrial fatty acid oxidation in the liver, we further pursued a possible role of SLC25A47 in the metabolism and transport of short-chain acyl-carnitines. To that end, we fed wild-type and *Slc25a47*^−/−^ mice HFD using either milk fat or mustard oil as the fat source. A high-fat diet enriched in medium-chain fatty acids (MCT oil), which supposedly do not require a transport protein for mitochondrial uptake, was used as a control. Milk fat is unique in that it contains branched-chain fatty acids [39]. Mustard oil is rich in the very long-chain fatty acid erucic acid (C22:1 n-9), which is dependent on peroxisomes for its oxidation [40]. The fatty acid composition of the diets is presented in Figure 10A and Table S2. The relative content of erucic acid in the mustard oil diet was 37.6%. Unfortunately, the GC analysis could not clearly separate and identify branched-chain fatty acids.

The type of HFD significantly influenced body weight. However, no differences in body weight were observed between wild-type and *Slc25a47*^−/−^ mice for any of the diets (Figure 10B). Similarly, no differences in weight of the gonadal fat pad were observed between wild-type and *Slc25a47*^−/−^ mice (Figure 10B). The diet also had a major influence on numerous liver and plasma parameters. In particular, milk fat raised liver weight and triglyceride content, as well as plasma levels of triglycerides, cholesterol, NEFA, and glycerol. By contrast, no significant effect of SLC25A47 deficiency on the above parameters was detected (Figure 10C). Next, we performed metabolomics analysis on liver tissue. Principal Component Analysis revealed a major overlap between wild-type and *Slc25a47*^−/−^ mice for all three diets (Figure 10D). Similarly, for all three diets, statistical analysis of the differences in metabolite levels between wild-type and *Slc25a47*^−/−^ mice resulted in a flat P-value distribution (Figure 10E). Interestingly, across all three diets, the comparison between wild-type and *Slc25a47*^−/−^ mice yielded only one metabolite that met the significance threshold of FDR < 0.05 or P < 0.0001, which was glutamate for the mustard oil diet (Figure 10F). Although less pronounced, plasma glutamate levels were also elevated in the *Slc25a47*^−/−^ mice on the milk fat and MCT oil diets (Figure 10G). Similar to the previous omics analyses, SLC25A47 deficiency was associated with changes in pathways related to the metabolism of amino acids and TCA cycle intermediates (Figure 10H, Sup Figure 5). Overall, these data do not suggest a role of SLC25A47 in the mitochondrial metabolism of very long-chain and branched fatty acids, yet do show an impact of SLC25A47 deficiency on hepatic glutamate levels, which interestingly was not observed in fasted mice. As in the previous HFD study, hepatic mRNA levels of macrophage markers and genes involved in liver fibrosis were sensitive to diet but were not influenced by SLC25A47 deficiency (Sup Figure 6). All in all, deficiency of SLC25A47 minimally impacted hepatic lipid metabolism in fasted and diet-induced obese mice.

## 4 Discussion

Here we identify *SLC25A47* as a novel PPARα-induced gene in mouse and human hepatocytes. To determine the physiological role of *SLC25A47*, we performed in vitro studies and conducted experiments in mice overexpressing or lacking *SLC25A47* under various dietary conditions. Overexpression of *Slc25a47* did not measurably impact metabolic parameters in mice fed a HFD or fasted for 24 h. Depending on the diet and fasting status, SLC25A47 deficiency was associated with statistically significant reductions in fasting plasma triglycerides and glycerol levels, improved glucose tolerance, and increased liver glutamate levels. The collective results are consistent with a largely redundant role of SLC25A47 in lipid and amino acid metabolism in the liver. Alternatively, it cannot be excluded that SLC25A47 mediates the mitochondrial transport of a major yet-to-be-identified metabolite. Our data do not confirm previously suggested roles of SLC25A47 in uncoupling, energy expenditure, lipid synthesis, fatty acid oxidation, liver fibrosis, mitochondrial stress, NAD^+^ transport, and gluconeogenesis.

We demonstrated that *SLC25A47* is a PPARα target gene. Interestingly, we found that *Slc25a4*7 mRNA in mouse liver is also induced by fasting in the absence of PPARα, which is reminiscent of fatty acid-sensitive genes such as *Plin5* and *Hilpda* [31]. Possibly, the induction of *Slc25a4*7 mRNA by fasting in PPARα^−/−^ mice is mediated by related nuclear receptors, potentially HNF4α, which was previously shown to cooperate with PPARα in the regulation of the *Gys2* gene [41], and was shown to bind to the *Slc25a47* gene [21].

Previously, it was reported that SLC25A47 functions as an uncoupling protein [17,18]. Uncoupled respiration describes the flow of electrons through the electron transport chain, the generation of a proton motive force, and the return of the protons to the mitochondrial matrix uncoupled from and without ATP synthesis [42]. In brown adipocytes, uncoupled respiration generates heat during cold exposure. It is critically dependent on uncoupling protein 1 (UCP1), a mitochondrial membrane transport protein that is part of the SLC25 family [43].

Whether the liver engages in uncoupled respiration is controversial. Tan and colleagues suggested that uncoupled respiration occurs in the liver and is mediated by SLC25A47 [17]. Studies in yeast cells confirmed the ability of SLC25A47 to uncouple mitochondrial respiration [18]. However, our studies in Hepa1-6 cells transiently and stably transfected with *Slc25a47* and in permeabilized livers from mice infected with *AAV-Slc25a47* or *AVV-GFP* do not support the role of SLC25A47 as an uncoupling protein. To what extent liver-specific uncoupling influences energy expenditure is unclear. In our studies in mice, SLC25A47 deficiency did not influence energy expenditure.

Recently, several papers were published investigating the role of SLC25A47 in the liver [[19], [20], [21]]. These studies were conducted concurrently with our work. Therefore, our analyses were not specifically set up to reproduce the results and validate the conclusions reached by others. For this reason, caution should be exercised when comparing the results obtained in the different papers. Nonetheless, below we have tried to relate our data to the outcomes from the other studies.

Bresciani et al. provided evidence that SLC25A47 is involved in mitochondrial homeostasis [19], while Yook et al. reported that SLC25A47 is involved in gluconeogenesis [21]. Both studies reported that male C57BL/6J *Slc25a47*^−/−^ mice have lower body weight and increased energy expenditure compared to wild-type littermates [19,21]. By contrast, we do not find an effect of SLC25A47 deficiency on body weight under numerous dietary conditions, including chow, LFD, different types of HFD, and after 24 h of fasting (in two independent large cohorts of mice). In addition, we did not find an effect of SLC25A47 deficiency on energy expenditure, independent of the time of the day and the feeding status.

According to Bresciani et al., hepatic deletion of *Slc25a47* impaired mitochondrial respiration, induced hepatic expression of genes involved in the mitochondrial stress response, exacerbated liver fibrosis, increased liver triglyceride content, and decreased liver glycogen content. In addition, both Bresciani et al. and Yook et al. observed a striking increase in serum FGF21 and hepatic *Fgf21* expression in *Slc25a47*^−/−^ mice [19,21]. The increase in energy expenditure and lower body weight were rescued when *Fgf21* was ablated on top of *Slc25a47*, suggesting that FGF21 is the driver for these metabolic effects [19], whereas changes in glucose tolerance, pyruvate tolerance, and mitochondrial respiration were independent of FGF21.

Our results do not confirm an increase in hepatic *Fgf2*1 mRNA in *Slc25a47*^−/−^ mice, as determined by qPCR and RNAseq. Also, other mitochondrial stress response genes were not affected by SLC25A47 deficiency. In fact, RNAseq revealed very limited changes in hepatic gene expression in SLC25A47 deficient mice, except for *Slc25a47* itself. Any changes in gene expression are likely to be indirect and may thus provide little insight into the specific function of SLC25A47. Likewise, we did not observe upregulation of fibrosis-related genes in *Slc25a47*^−/−^ mice fed different types of HFD. Furthermore, we consistently did not find a significant effect of SLC25A47 deficiency on liver triglyceride or glycogen content. Besides SLC25A47 deficiency, SLC25A47 overexpression also did not influence liver triglyceride content, either in fasted mice or mice fed HFD. This is in contrast with the results of Cheng and colleagues, who reported reduced liver triglycerides in mice overexpressing SLC25A47 [20].

Deficiency of SLC25A47 was reported to be associated with improved insulin sensitivity and reduced hepatic gluconeogenesis, concurrent with lower blood glucose levels [19,21]. Although in our study SLC25A47 deficiency was associated with improved glucose tolerance in mice fed HFD, plasma glucose levels remained unaltered in numerous dietary conditions investigated. Metabolomics on plasma, whole liver, and liver mitochondria did not reveal any changes in levels of gluconeogenic substrates by ablation of SLC25A47. Currently, it is unclear if the improved glucose homeostasis observed by Yook and colleagues is coupled with the reduced body weight and adiposity of the *Slc25a47*^−/−^ mice. Ideally, clamp studies should be conducted to determine the impact of SLC25A47 deficiency on hepatic glucose production and peripheral glucose disposal.

Previously, SLC25A51, which is related to SLC25A47, was identified as a mitochondrial NAD^+^ transporter. *Slc25a51* deficiency was shown to alter TCA metabolite abundance, lower the respiratory chain capacity, and reduce ATP production, along with depleting mitochondrial NAD^+^ levels [44,45]. Recently, Cheng et al. proposed that SLC25A47 is also an NAD^+^ mitochondrial transporter [20]. In our study, we did not directly measure NAD^+^ levels, nor was NAD^+^ part of the metabolite panel in the metabolomics analysis. Overall, however, our study very poorly phenocopied the data by Cheng on SLC25A47 overexpression and deficiency.

While most of our measurements did not show any effect of SLC25A47 overexpression and deficiency, we consistently found reduced fasting plasma triglyceride levels in *Slc25a47*^−/−^ mice. Across all animals studied in the 24-hour fasted state, plasma triglyceride levels were about 20% lower in *Slc25a47*^−/−^ mice, whereas liver triglyceride content was not affected (Sup Figure 7). Bresciani et al. did not measure plasma triglycerides in *Slc25a47*^−/−^ mice. Yook et al. only studied the non-fasted state and did not find an effect of *Slc25a47* ablation on plasma triglyceride levels, whereas Cheng et al. observed increased plasma triglycerides in *Slc25a47*^−/−^ mice and decreased plasma triglycerides upon SLC25A47 overexpression. Based on our analysis, the decreased plasma triglycerides in *Slc25a47*^−/−^ mice are not related to a decrease in hepatic triglyceride production or secretion. It would be interesting to determine whether triglyceride clearance and uptake by extrahepatic tissues are increased following *Slc25a47* ablation, for example by studying plasma triglyceride clearance using radiolabeled VLDL-like emulsion particles [46].

Overall, no consistent picture of the function of SLC25A47 emerges from the detailed assessment of the various papers. Vastly different metabolic outcomes in the *Slc25a47*^−/−^ mice are reported across the different papers, with our paper showing the mildest phenotype. Perhaps the most striking difference is that Bresciani et al. and Yook et al. already noticed differences in body weight and length between wild-type and *Slc25a47*^*−/−*^ mice on a normal chow diet, even without any additional intervention [19,21].

Importantly, while our paper was under review, a paper was published [47] that provides an explanation for the discrepancies between our paper and the papers by Bresciani et al. [19] and Yook et al. [21]. Although Bresciani et al. and Yook et al. acquired the same *Slc25a47*-mutant mice (Slc25a47tm1a(EUCOMM)Hmgu) from the EUCOMM/KOMP repository as used in our studies, they used a different approach to genetically inactivate the *Slc25a47* gene. Specifically, Bresciani et al. [19] and Yook et al. [21] generated *Slc25a47* Tm1c mice, which were crossed with Albumin-Cre mice to generate *Slc25a47* Tm1d mice. However, this strategy inadvertently deletes a transcription termination signal, resulting in the production of a chimeric RNA composed of truncated *Slc25a47* sense RNA fused to anti-sense *Wars1*, a gene that is located ∼3 kb downstream of the *Slc25a47* gene. Alongside the loss of *Slc25a47*, this leads to the hepatocyte-specific downregulation of *Wars1*. In contrast, the generation of Tm1a/Tm1b mice does not create the same issue and does not lead to the simultaneous downregulation of *Wars1*, as confirmed by qPCR. Crucially, it was shown that the observed effects on mitochondrial stress response and energy expenditure in the Tm1d mice used by Bresciani et al. are driven by the downregulation of *Wars1*, rather than SLC25A47 deficiency [47]. These findings invalidate the previous conclusions reached using Tm1d mice that SLC25A47 regulates mitochondrial energy homeostasis and corroborate the limited phenotype associated with SLC25A47 deficiency in our study.

Our paper has limitations. An important limitation is that we were unable to demonstrate the absence of SLC25A47 protein in the *Slc25a47*^−/−^ mice. Despite investing a huge effort and significant resources, we were unable to obtain trustworthy results. Nonetheless, since both qPCR and RNAseq showed a near-total absence of *Slc25a4*7 mRNA, which was observed in both Tm1a and Tm1b groups, we are confident that we have a true knockout. Another limitation is that most of our studies were done in *Slc25a47*^−/−^ mice on a mixed C57BL/6J and C57BL/6N background. However, this is unlikely to account for the lack of a prominent phenotype in the *Slc25a47*^−/−^ mice. Moreover, we validated our key findings in wild-type and *Slc25a47*^−/−^ littermates on a pure C57BL/6J background. Finally, we did not do actual transport studies, since our results did not provide good candidate substrates. Transport studies require very specific expertise and could be envisioned as a potential future research direction. The strength of our paper is that we use relatively large numbers of mice for all of our experiments and that we subjected two independent large cohorts of wild-type and *Slc25a47*^−/−^ mice to fasting and high-fat feeding, leading to highly similar outcomes.

Taken together, we demonstrate that *SLC25A47* is a novel PPARα-regulated gene in human and mouse hepatocytes. Apart from modest reductions in fasting plasma triglycerides and glycerol, our studies do not reveal a pronounced metabolic phenotype in *Slc25a47*^−/−^ mice. Recognizing that our studies were not designed to specifically validate the outcomes from other papers, we nonetheless conclude that we fail to confirm the suggested role of SLC25A47 in uncoupling, energy expenditure, fatty acid oxidation, mitochondrial stress, liver fibrosis, gluconeogenesis, fatty acid and cholesterol synthesis, and NAD^+^ transport. Overall, we conclude that SLC25A47 is dispensable for hepatic lipid homeostasis during fasting and high-fat feeding.

## Funding sources

Funding from the Netherlands Organisation for Scientific Research (2014/12392/ALW), Consejo Nacional de Ciencia y Tecnologia de México (CONACYT-455071), and the Netherlands Cardiovascular Research Initiative (CVON2014-02 ENERGISE), an initiative with the support of the Dutch Heart Foundation, is gratefully acknowledged.

## CRediT authorship contribution statement

**Brecht Attema:** Writing – review & editing, Writing – original draft, Visualization, Methodology, Investigation, Formal analysis, Data curation, Conceptualization. **Montserrat A. de la Rosa Rodriguez:** Writing – review & editing, Writing – original draft, Visualization, Methodology, Investigation, Formal analysis, Data curation, Conceptualization. **Evert M. van Schothorst:** Writing – review & editing, Methodology, Formal analysis, Conceptualization. **Sander Grefte:** Writing – review & editing, Methodology, Formal analysis, Conceptualization. **Guido JEJ. Hooiveld:** Writing – review & editing, Formal analysis, Data curation. **Sander Kersten:** Writing – review & editing, Writing – original draft, Visualization, Supervision, Project administration, Methodology, Investigation, Funding acquisition, Formal analysis, Conceptualization.

## Declaration of competing interest

The authors declare that they have no known competing financial interests or personal relationships that could have appeared to influence the work reported in this paper.

## Data Availability

Data will be made available on request.
