# Supplementary material for: Deficiency of the mitochondrial transporter SLC25A47 minimally impacts hepatic lipid metabolism in fasted and diet-induced obese mice
Source: Mol Metab. 2024 Dec 31;92:102092. doi: 10.1016/j.molmet.2024.102092 (PMC11773045; doi:10.1016/j.molmet.2024.102092)
Supplement: Multimedia component 1 [file mmc1.pdf]

Supplementary Figures of "Deficiency of the mitochondrial transporter SLC25A47 minimally impacts hepatic lipid metabolism in fasted and diet-induced obese mice"

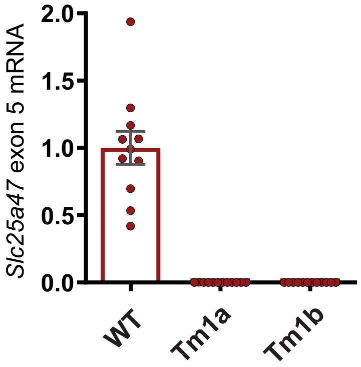

Supplementary Figure 1. mRNA expression of *Slc25a47* in livers of male wild type, Tm1a and Tm1b mice. Mice were fed a low-fat diet for 20 weeks. Primers were directed against exon 5 of the *Slc25a47* gene.

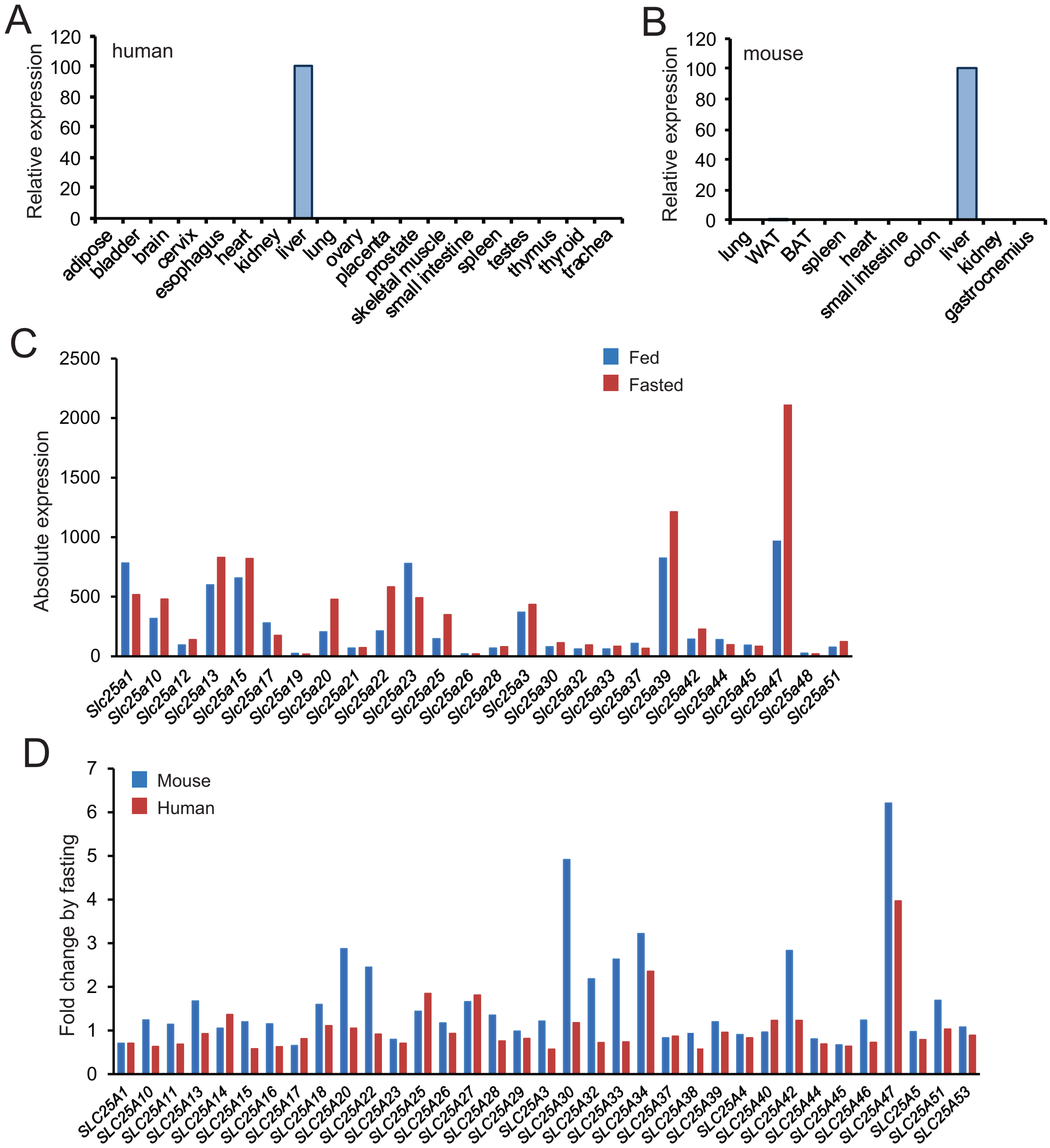

Supplementary Figure 2. A) Expression of *SLC25A47* mRNA in various human tissues as determined by qPCR. Human RNA was a mix of several individuals (AMBION, First Choice human total RNA). B) Expression of *Slc25a47* mRNA in various mouse tissues as determined by qPCR. Mouse RNA came from one healthy female adult mouse (strain FVB). Expression levels were related to the tissue showing the highest expression. C) Expression of members of the *Slc25* gene family in livers of ad libitum fed and 16h fasted mice according to transcriptome analysis (GSE156254). D) Expression of members of the *Slc25* gene family in livers of 24h fasted hepatocyte-humanized mice according to transcriptome analysis. The human and mouse transcriptomes were analyzed separately (GSE126587).

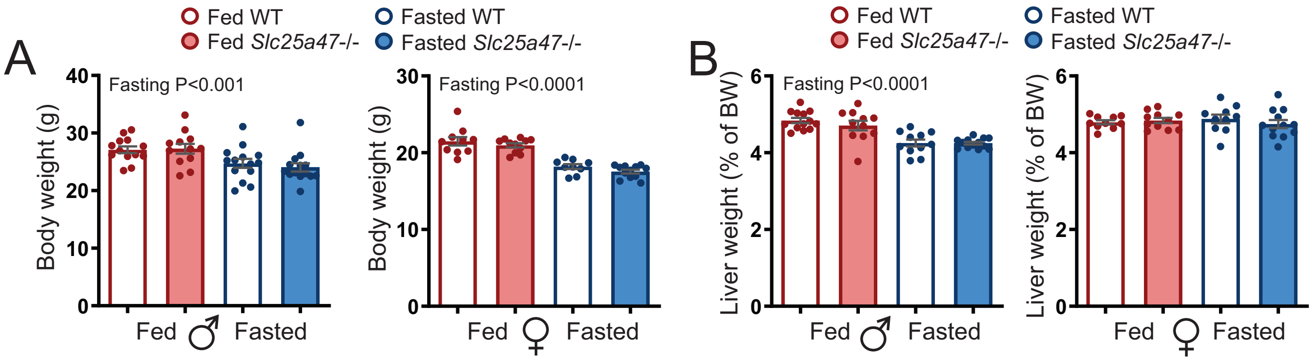

Supplementary Figure 3. Body weight and liver weight in male and female wild-type and *Slc25a47*–/– mice in the ad libitum fed state or after 24h of fasting (N=10-14 mice per group). The wild-type and *Slc25a47*–/– mice were littermates on a pure C57BL/6J background and obtained via heterozygous breeding. Data were analyzed by two-way ANOVA.

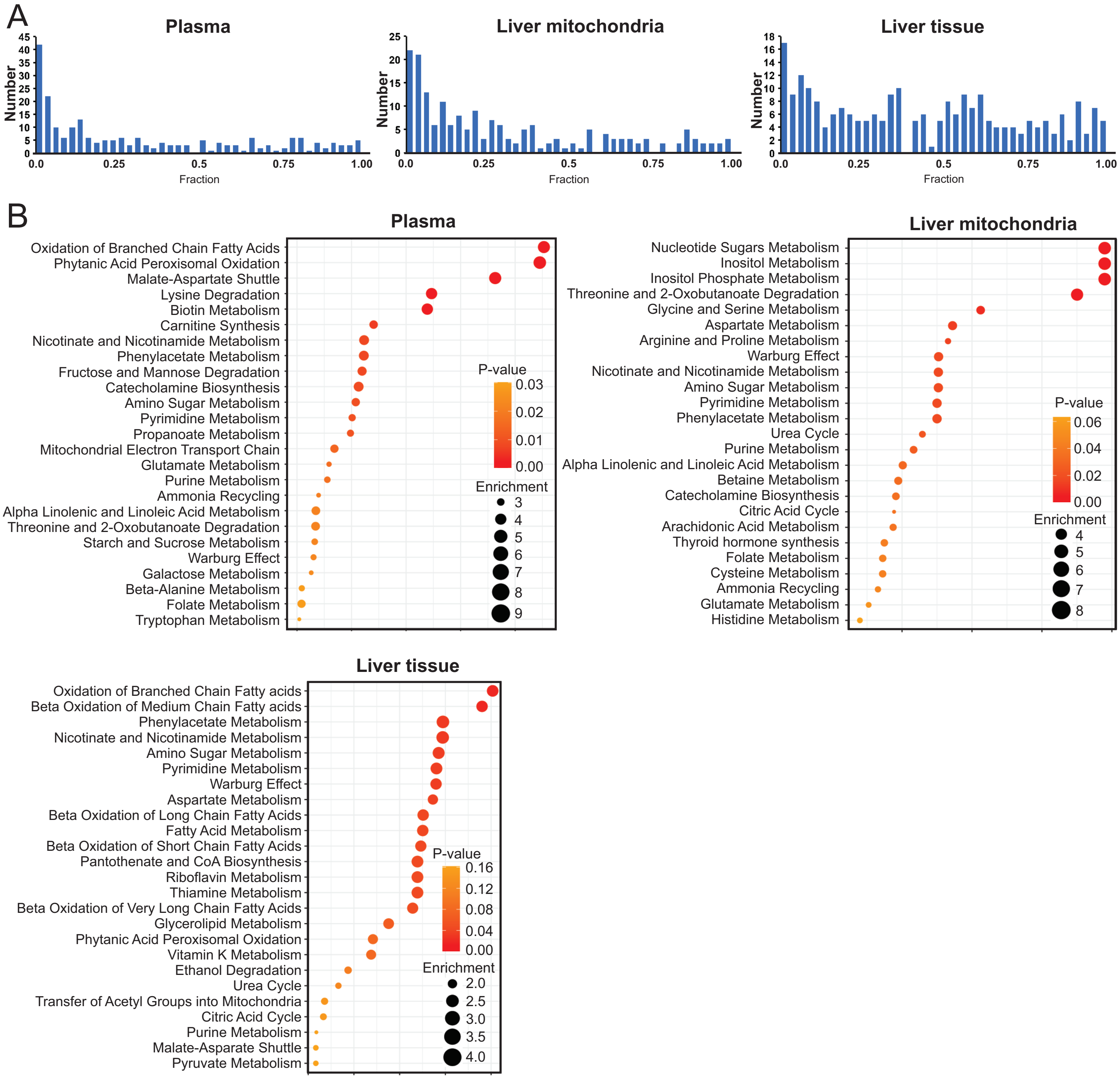

Supplementary Figure 4. A) P-value distribution of metabolome analysis comparing plasma, liver mitochondria, or liver tissue from fasted wild-type and *Slc25a47–/–* mice (N=8/group). B) Overview of Enriched Metabolite Sets (Top 25) comparing plasma, liver mitochondria, or liver tissue metabolome data from 24h fasted wild-type and *Slc25a47–/–* mice. The wild-type and *Slc25a47–/–* mice were not littermates and were obtained via parallel breeding (mixed C57BL/6J and C57BL/6N background).

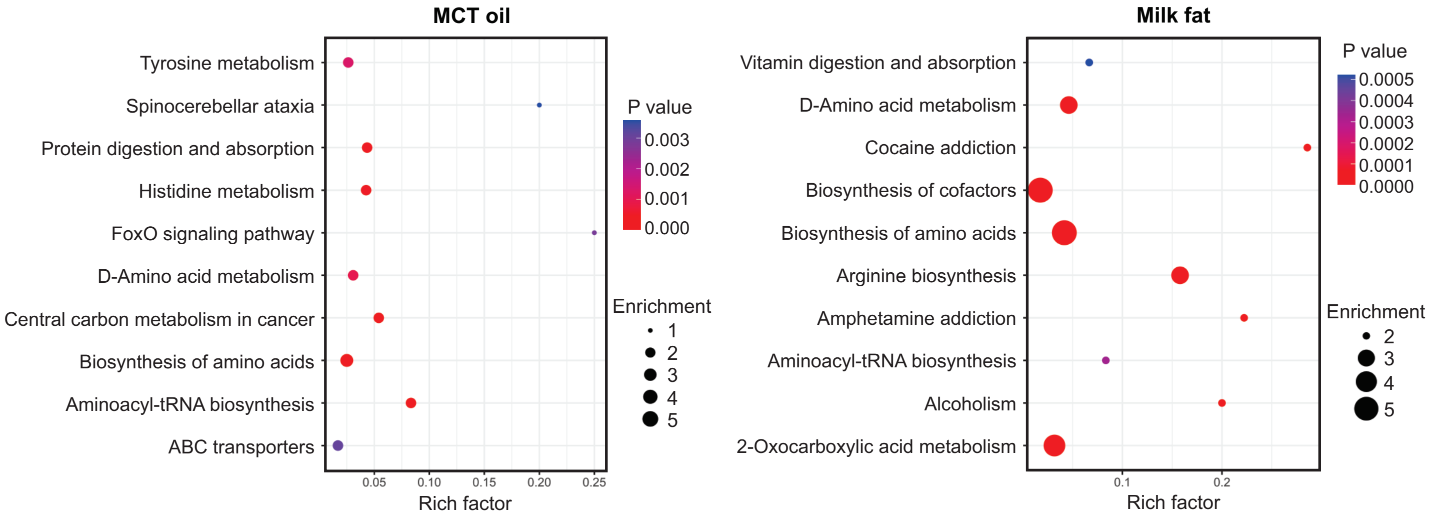

Supplementary Figure 5. Overview of Enriched Metabolite Sets (Top 10) comparing liver metabolome data from wild-type and *Slc25a47*–/– mice fed high-fat diets enriched in MCT oil or milk fat. The wild-type and *Slc25a47*–/– mice were not littermates and were obtained via parallel breeding (mixed C57BL/6J and C57BL/6N background).

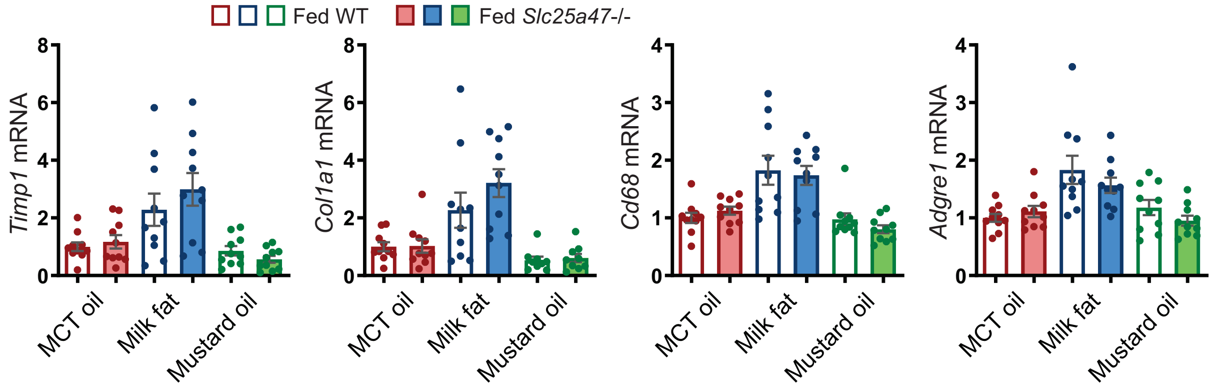

Supplementary Figure 6. Expression of fibrosis and inflammatory marker genes in livers of wild-type and *Slc25a47*–/– mice fed high-fat diets enriched in MCT oil, milk fat, or mustard oil (N=10 per group). The wild-type and *Slc25a47*–/– mice were not littermates and were obtained via parallel breeding (mixed C57BL/6J and C57BL/6N background).

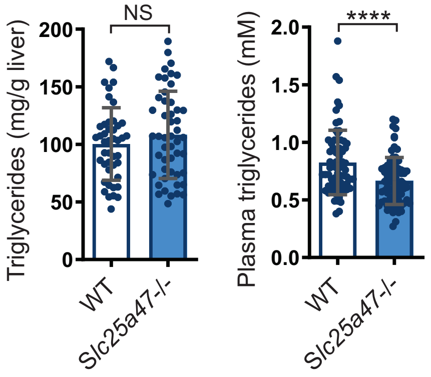

Supplementary Figure 7. Left: Combined liver triglyceride levels from 24h fasted wild-type (N=44) and *Slc25a47–/–* (N=49) mice across several independent studies. Right: Combined plasma triglyceride levels from 24h fasted wild-type (N=72) and *Slc25a47–/–* (N=77) mice across several independent studies. Data were analyzed by Student's t-test (****P<0.0001).
