# Supplementary material for: Deficiency of the mitochondrial transporter SLC25A47 minimally impacts hepatic lipid metabolism in fasted and diet-induced obese mice
Source: Mol Metab. 2024 Dec 31;92:102092. doi: 10.1016/j.molmet.2024.102092 (PMC11773045; doi:10.1016/j.molmet.2024.102092)
Supplement: Multimedia component 2 [file mmc2.docx]

**Supplementary Tables of “Deficiency of the mitochondrial transporter SLC25A47 minimally impacts hepatic lipid metabolism in fasted and diet-induced obese mice”**

Table S1: Macronutrient and ingredient composition of the various diets

|  | ***HFD MCT oil*** | | ***HFD Milk fat*** | | ***HFD Mustard oil*** | |
| --- | --- | --- | --- | --- | --- | --- |
| **Based on formula #** | **D12451** | | **D12451** | | **D12451** | |
|  | **g%** | ***kcal%*** | **g%** | ***kcal%*** | **g%** | ***kcal%*** |
| Protein | 24 | 20 | 24 | 20 | 24 | 20 |
| Carbohydrate | 41 | 35 | 41 | 35 | 41 | 35 |
| Fat | 24 | 45 | 24 | 45 | 24 | 45 |
| Total |  | 100 |  | 100 |  | 100 |
| kcal/g | 4.7 |  | 4.7 |  | 4.7 |  |
|  |  |  |  |  |  |  |
| **Ingredient** | **g** | **kcal** | **g** | **kcal** | **g** | **kcal** |
| Casein, lactic | 200 | 800 | 200 | 800 | 200 | 800 |
| L-Cystine | 3 | 12 | 3 | 12 | 3 | 12 |
|  |  |  |  |  |  |  |
| Corn Starch | 72.8 | 291 | 72.8 | 291 | 72.8 | 291 |
| Maltodextrin 10 | 100 | 400 | 100 | 400 | 100 | 400 |
| Sucrose | 172.8 | 691 | 172.8 | 691 | 172.8 | 691 |
| Cellulose, BW200 | 50 | 0 | 50 | 0 | 50 | 0 |
|  |  |  |  |  |  |  |
| Soybean Oil | 25 | 225 | 25 | 225 | 25 | 225 |
| Butter fat | 0 | 0 | 177.5 | 1598 | 0 | 0 |
| Mustard oil | 0 | 0 | 0 | 0 | 177.5 | 1598 |
| MCT oil | 177.5 | 1598 | 0 | 0 | 0 | 0 |
|  |  |  |  |  |  |  |
| Mineral Mix S10026 | 10 | 0 | 10 | 0 | 10 | 0 |
| DiCalcium Phosphate | 13 | 0 | 13 | 0 | 13 | 0 |
| Calcium Carbonate | 5.5 | 0 | 5.5 | 0 | 5.5 | 0 |
| Potassium Citrate | 16.5 | 0 | 16.5 | 0 | 16.5 | 0 |
| Vitamin Mix V10001 | 10 | 40 | 10 | 40 | 10 | 40 |
| Choline Bitartrate | 2 | 0 | 2 | 0 | 2 | 0 |
|  |  |  |  |  |  |  |
| FD&C Yellow Dye #5 | 0.025 | 0 | 0.025 | 0 | 0 | 0 |
| FD&C Red Dye #40 | 0.025 | 0 | 0 | 0 | 0 | 0 |
| FD&C Blue Dye #1 | 0 | 0 | 0.025 | 0 | 0.05 | 0 |
| **Total (grams)** | **858.15** | **4057** | **858.15** | **4057** | **858.15** | **4057** |

Table S2: Fatty acid composition of the various diets

|  | ***MCT oil*** | ***Mustard oil*** | ***Milk fat*** |
| --- | --- | --- | --- |
| **C8:0** | 49.23 | 0.04 | 0.75 |
| **C10:0** | 30.32 | 0.07 | 1.99 |
| **C11:0** | 0.09 | 0 | 0.07 |
| **C12:0** | 0.14 | 0.03 | 2.62 |
| **C13:0** | 0.03 | 0.02 | 0.11 |
| **C14:0** | 0.11 | 0.13 | 8.69 |
| **C14:1** | 0.03 | 0.03 | 0.72 |
| **C15:0** |  | 0.02 | 0.98 |
| **C16:0** | 2.16 | 3.4 | 29.81 |
| **C16:1 n-7** | 0.03 | 0.22 | 1.32 |
| **C16:1 n-9** |  | 0.03 | 0.13 |
| **C16:2 n-4** |  | 0.03 | 0.03 |
| **C16:3 n-3** |  | 0.08 |  |
| **C17:0** | 0.04 | 0.05 | 0.58 |
| **C18:0** | 0.81 | 1.59 | 10.77 |
| **C18:1 n-7** | 0.3 | 1.33 | 2.07 |
| **C18:1 n-9** | 3.82 | 10.68 | 20.41 |
| **C18:2 n-11t/9c** | 0.03 | 0.02 | 0.33 |
| **C18:2 n-6** | 9.39 | 19.34 | 10.37 |
| **C18:3 n-3** | 1.15 | 8.86 | 1.31 |
| **C18:3 n-6** |  |  | 0.02 |
| **C19:0** |  | 0.02 | 0.08 |
| **C20:0** | 0.07 | 0.94 | 0.19 |
| **C20:1 n-12 or n-11** |  |  | 0.12 |
| **C20:1 n-7** |  | 1.77 | 0.05 |
| **C20:1 n-9** | 0.06 | 4.76 | 0.08 |
| **C20:2 n-6** | 0.03 | 0.69 | 0.06 |
| **C20:3 n-3** |  | 0.13 |  |
| **C20:3 n-6** |  |  | 0.11 |
| **C20:4 n-3** |  |  | 0.03 |
| **C20:4 n-6** |  | 0.03 | 0.15 |
| **C20:5 n-3** | 0.03 | 0.03 | 0.04 |
| **C21:0** |  | 0.03 | 0.03 |
| **C22:0** | 0.08 | 1.26 | 0.1 |
| **C22:1 n-11** | 0.02 |  | 0.03 |
| **C22:1 n-9** | 0.03 | 37.64 | 0.08 |
| **C22:2 n-6** | 0.03 | 1.24 |  |
| **C22:3 n-3** | 0.69 | 0.67 | 0.4 |
| **C22:4 n-6** | 0.37 | 0.03 | 0.26 |
| **C22:5 n-3** | 0.03 | 0.05 | 0.07 |
| **C22:5 n-6** | 0.03 | 0.01 |  |
| **C22:6 n-3** |  |  |  |
| **C23:0** | 0.03 | 0.06 | 0.04 |
| **C24:0** | 0.04 | 0.73 | 0.04 |
| **C24:1 n-9** |  | 1.85 |  |
